# Chemical and biological characterization of glycidamide-adducted adenine in DNA

**DOI:** 10.1016/j.jbc.2025.110898

**Published:** 2025-11-04

**Authors:** Jun-ichi Akagi, Ryota Yamaguchi, Yumi Miyake, Masayuki Yokoi, Kaoru Sugasawa, Shigenori Iwai

**Affiliations:** 1 Division of Pathology, National Institute of Health Sciences, Kawasaki, Kanagawa, Japan; 2 Division of Chemistry, Graduate School of Engineering Science, Osaka University, Toyonaka, Osaka, Japan; 3 Forefront Research Center, Graduate School of Science, Osaka University, Toyonaka, Osaka, Japan; 4 Core Facility Center, Osaka University, Toyonaka, Osaka, Japan; 5 Biosignal Research Center, Kobe University, Kobe, Hyogo, Japan; 6 Frontiers of Innovative Research in Science and Technology, Konan University, Kobe, Hyogo, Japan

**Keywords:** DNA lesion, mutagenesis, DNA replication, nucleic acid chemistry, acrylamide, glycidamide, *N*^6^-adducted adenine

## Abstract

Acrylamide, a food contaminant, is metabolically converted to glycidamide (GA), which reacts with nucleobases in DNA. Although several GA adducts with dG, 2′-deoxyadenosine (dA), and dC are known, the mutagenic potency of each adduct remains unclear. Here, we focused on *N*^6^-(2-carboxy-2-hydroxyethyl)-2′-deoxyadenosine (*N*^6^-GA-dA), which is produced by the Dimroth rearrangement of N1-(2-carboxy-2-hydroxyethyl)-2′-deoxyadenosine. We separately prepared N1-(2-carboxy-2-hydroxyethyl)-2′-deoxyadenosine and *N*^6^-GA-dA and measured their Dimroth rearrangement reaction rates. Since *N*^6^-GA-dA is the stable final product under physiological conditions (pH 7.0, at 37°C), we examined its presence in genomic DNA isolated from GA-treated cells. *N*^6^-GA-dA was successfully detected by LC-MS analysis of the nucleoside components of DNA from XP2OSSV cells, at about 40 adducts per 10^8^ dA. In addition, *N*^6^-(2-deoxy-d-*erythro*-pentofuranosyl)-2,6-diamino-3,4-dihydro-4-oxo-5-[*N*-(2-carbamoyl-2-hydroxyethyl)formamido]pyrimidine, which we have previously shown its mutagenic potency, was also detected at 5 × 10^4^ adducts per 10^8^ dG, a level comparable to N7-(2-carbamoyl-2-hydroxyethyl)guanine. To assess the biological impact of *N*^6^-GA-dA, we prepared oligonucleotides containing a single *N*^6^-GA-dA at a specific position and analyzed base-pair formation and mutagenic potential. The thermodynamic parameters of duplexes revealed that *N*^6^-GA-dA forms a base pair with thymidine similarly to dA. DNA polymerase ε bypassed *N*^6^-GA-dA by incorporating the correct dTTP opposite the lesion. Furthermore, a site-specific intracellular mutagenesis assay showed no detectable replication inhibition or mutation induction at the lesion site. In summary, we detected and quantified *N*^6^-GA-dA formed in GA-treated cells and investigated its mutagenic potency. This study contributes to understanding the molecular aspects of GA adducts in DNA.

Acrylamide (AA) is a chemical substance widely used for manufacturing various industrial products in the form of its polymer, polyacrylamide. AA is also found in cooked foods, such as potato crisps and French fries, and is primarily produced by the Maillard reaction between asparagine and a reducing sugar at high temperature (1, 2). When AA is ingested, it is metabolized to glycidamide (GA) by cytochrome P450 2E1 oxidation (3). Since GA contains oxacyclopropane, a three-membered heterocyclic ring, it reacts with nucleophiles in biomolecules.

In DNA, a well-recognized GA reaction site is N7 of guanine. Since the N7 adduct of 2′-deoxyguanosine (dG) is labile because of the positive charge on the nitrogen atom, its glycosidic bond is easily cleaved, and N7-(2-carbamoyl-2-hydroxyethyl)guanine (N7-GA-Gua) is released from the DNA strand (4). Recently, we showed that *N*^6^-(2-deoxy-_D_-*erythro*-pentofuranosyl)-2,6-diamino-3,4-dihydro-4-oxo-5-[*N*-(2-carbamoyl-2-hydroxyethyl)formamido]pyrimidine (GA-FAPy-dG) remains in DNA and is formed by the hydrolytic ring opening of the N7-GA adduct of dG (5). Another recent study found that the formation of this FAPy derivative is characteristic of GA, whereas the glycosidic bond cleavage is predominant in the cases of the N7 adducts of other oxacyclopropane-containing compounds, such as glycidol and 1,2-epoxybutane (6).

Various GA reactions with other nucleosides have been reported. For 2′-deoxyadenosine (dA), two major products, N1-(2-carboxy-2-hydroxyethyl)-2′-deoxyadenosine (N1-GA-dA) and *N*^6^-(2-carboxy-2-hydroxyethyl)-2′-deoxyadenosine (*N*^6^-GA-dA), in which the carbamoyl group in the GA moiety was hydrolyzed to a carboxy group, were detected (7, 8), in addition to N3-(2-carbamoyl-2-hydroxyethyl)adenine (N3-GA-Ade) released from DNA strands (7). The structures of the two nucleosides are shown in Figure 1*A*. *N*^6^-GA-dA is produced by the Dimroth rearrangement of N1-GA-dA (7, 8), and a small amount of N1-(2-carboxy-2-hydroxyethyl)-2′-deoxyinosine, produced by the hydrolysis of the amino group, was also reported when dA was treated with GA (8). Regarding the cytosine base, the reaction of GA with cytidine, a ribonucleoside, primarily produced N3-(2-carboxy-2-hydroxyethyl)cytidine, containing a carboxy group generated by hydrolysis of the carbamoyl group (9). Adduct formation with thymidine occurs at pH 9.0 and yields N3-(2-carbamoyl-2-hydroxyethyl)thymidine, but substantially lower amounts are obtained at pH 7 (6, 9).

**Figure 1:**
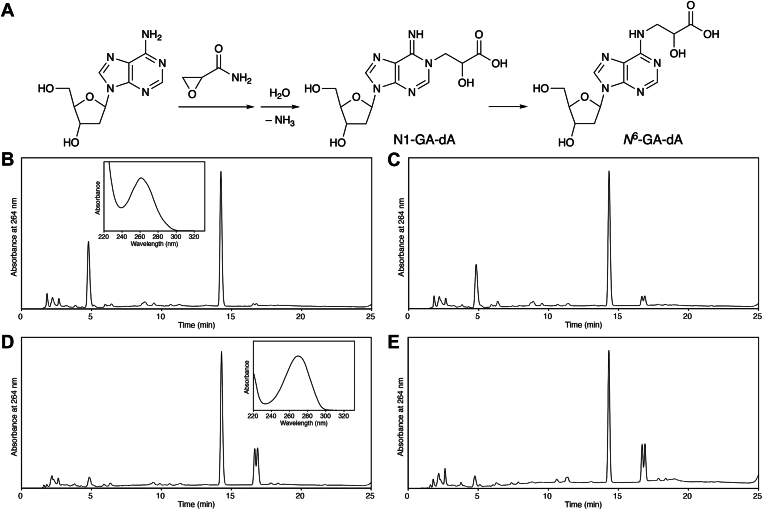
**Adduct formation of GA with dA.***A*, reaction of GA with dA. *B*–*D*, HPLC analyses of reaction mixtures containing dA and GA in 0.1 M sodium phosphate (pH 6.0 [*B*], pH 7.0 [*C*], or pH 8.0 [D]) after an incubation at 37 °C for 72 h. Chromatograms monitored at 264 nm are shown to compare product amounts. The largest peak at 14.3 min is the starting material, dA. The products at 4.8 min (*B*) and 16.7 and 16.9 min (*D*), which had absorption maxima at 260 and 268 nm (*insets*), were assigned to N1-GA-dA and *N*^6^-GA-dA, respectively. *E*, HPLC analysis of a reaction mixture containing dA and GA in 0.1 M sodium phosphate (pH 7.0) after incubation at 60 °C for 24 h. dA, 2′-deoxyadenosine; GA, glycidamide.

Due to the reactivity of the oxacyclopropane ring, GA is considered to cause the genotoxicity and carcinogenicity of AA (10). AA is classified as group 2A (probably carcinogenic to humans) by the International Agency for Research on Cancer. Several studies have assessed the mutational signatures of AA and GA. Analyses of the mutation spectra in mouse embryonic fibroblasts exposed to GA revealed T:A > A:T and C:G > A:T transversions and T:A > C:G transitions (11, 12), and a recent study reported that the A:T > G:C mutation in the nAt sequence motif occurred most frequently in yeast single-stranded DNA (13). However, the molecular mechanism by which GA causes these mutations has not been elucidated. As a rare example, we previously showed that GA-FAPy-dG mainly induces G:C > A:T transitions, likely because of the misincorporation of dTTP by DNA polymerase κ (5).

In this study, we focused on the adduct formation of GA with dA. We first confirmed the formation of N1-GA-dA and *N*^6^-GA-dA and analyzed their Dimroth rearrangement reactions. Next, we established the presence of the stable product *N*^6^-GA-dA in genomic DNA isolated from GA-treated cells by LC-MS analyses of the nucleoside components. Two guanine adducts, N7-GA-Gua and GA-FAPy-dG, whose structures are shown in Fig. S1, were also detected in the same experiments. We then investigated base-pair formation and the mutagenic potency of *N*^6^-GA-dA using oligonucleotides containing this adduct at a single position.

## Results

### Formation of N1-GA-dA and *N*^6^-GA-dA

The reaction between GA and dA was analyzed first. dA was mixed with an excess amount of GA in pH 6.0, 7.0, and 8.0 buffers. These mixtures were incubated at 37 °C for 72 h, and subsequently aliquots were analyzed by reversed-phase HPLC. After the reaction at pH 6.0, a product peak with a UV absorption maximum at 260 nm was detected at a very short retention time (Fig. 1*B*). At pH 8.0, two product peaks were observed at longer retention times than that of dA (Fig. 1*D*). The two products obtained at pH 8.0 were presumed to be the stereoisomers at the carbon atom bearing the hydroxy group in the GA moiety based on their identical UV-absorption spectra (λ_max_ = 268 nm). At pH 7.0, both types of products were formed (Fig. 1*C*), and when the mixture of GA and dA in pH 7.0 buffer was incubated at 60 °C for 24 h, the products obtained at pH 8.0 were detected predominantly (Fig. 1*E*). In LC-MS analyses, the *m*/*z* values of [M + H]^+^ obtained for the products at pH 6.0 and pH 8.0 were both 340.125. Two types of GA adducts, N1-GA-dA and *N*^6^-GA-dA, in which the carbamoyl group in the GA moiety is hydrolyzed to a carboxy group (Fig. 1*A*), are produced by the reaction of GA with dA at 37 °C and pH 7.0 (8). The calculated *m*/*z* value of [M + H]^+^ of these compounds is 340.1252. The LC-MS results indicated that each reaction resulted in the formation of one of these adducts. From the reported UV-absorption spectra (8), the products formed at pH 6.0 and pH 8.0 were assigned to N1-GA-dA and *N*^6^-GA-dA, respectively. In the chromatogram of the pH 7.0 reaction at 60 °C (Fig. 1*E*), very small doublet peaks with retention times of 11.3 and 11.4 min were assigned to the stereoisomers of N1-(2-carboxy-2-hydroxyethyl)-2′-deoxyinosine, produced by hydrolysis of the amino group in the adenine base, as determined by the UV-absorption spectra (8).

**Figure 2:**
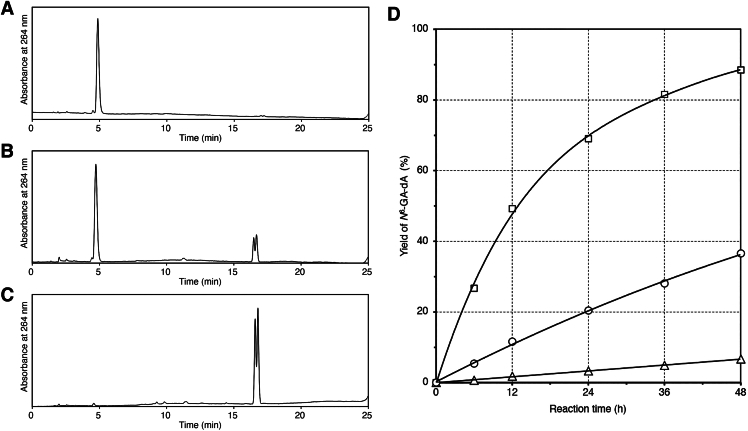
**Dimroth rearrangement from N1-GA-dA to *N*^6^-GA-dA.***A*–*C*, HPLC analyses of isolated N1-GA-dA (*A*), a pH 7.0 solution of N1-GA-dA incubated at 37 °C for 24 h (*B*), and a pH 8.0 solution of N1-GA-dA incubated at 60 °C for 24 h (*C*). *D*, time courses of the rearrangement reaction at pH 6.0 (*triangles*), 7.0 (*circles*), and 8.0 (*squares*) solutions at 37 °C. N1-GA-dA, N1-(2-carboxy-2-hydroxyethyl)-2′-deoxyadenosine; *N*^6^-GA-dA, *N*^6^-(2-carboxy-2-hydroxyethyl)-2′-deoxyadenosine.

*N*^6^-GA-dA is reportedly generated by the Dimroth rearrangement of N1-GA-dA (7, 8). We investigated this reaction to confirm the structures of the obtained products and the reaction mechanism. N1-GA-dA was prepared by the reaction at pH 5.0, isolated by HPLC purification, and incubated in pH 7.0 buffer at 37 °C for 24 h. The HPLC analysis clearly showed the rearrangement of N1-GA-dA to *N*^6^-GA-dA (Fig. 2, *A* and *B*). N1-GA-dA was completely converted to *N*^6^-GA-dA when its solution in pH 8.0 buffer was incubated at 60 °C for 24 h (Fig. 2*C*). To understand this reaction in more depth, time-course experiments at 37 °C were performed in solutions with different pH values. As shown in Figure 2*D*, the reaction was faster at higher pH, and the rate constants for the reactions at pH 6.0, 7.0, and 8.0 were calculated to be 1.4 × 10^−^^3^, 9.4 × 10^−^^3^, and 4.7 × 10^−^^2^ h^−^^1^, respectively.

### Detection of the adducts in genomic DNA isolated from GA-treated cells

To determine whether *N*^6^-GA-dA is actually formed in cellular DNA, we analyzed the nucleoside components of genomic DNA isolated from GA-treated cells by LC-MS (14). Using the same DNA sample, we also tried to detect GA-FAPy-dG and N7-GA-Gua, which we studied previously (5). Prior to the analysis, the standard compounds were prepared, as described in the *Experimental procedures* section. HPLC elution profiles and UV absorption spectra of these three compounds are shown in Fig. S1, and their separation from the canonical nucleosides on a semipreparative column was confirmed (Fig. S2, *A* and *B*).

For the detection of chemically stable GA adducts existing in genomic DNA, we used nucleotide excision repair (NER)-deficient XP2OSSV cells to reduce the possibility of GA adduct removal from DNA during the treatment period. Although it remains unclear whether NER actually participates in the removal of GA adducts, global genome NER recognizes DNA helices containing damaged bases with a large alteration in chemical structure and therefore can remove a variety of DNA lesions (15), whereas transcription-coupled NER removes lesions on actively transcribed strands. XP2OSSV cells have a defect in the XPA protein, which is essential for both global genome NER and transcription-coupled NER. Therefore, we hypothesized that using XP2OSSV cells would improve the detectability of the GA adducts in genomic DNA. Prior to the preparation of genomic DNA, we optimized the conditions for GA treatment. When XP2OSSV cells were treated with 1 mM GA for 24 h, cell viability decreased to about 50% compared with untreated cells (Fig. S3*A*), whereas no obvious morphological changes were observed by phase-contrast microscopy (Fig. S3*B*, *upper panels*). However, the cells were severely damaged at 48 h after treatment (Fig. S3*B*, *lower panels*), indicating that an intolerable amount of GA adducts was produced by this GA treatment. Based on these results, XP2OSSV cells were treated with 1 mM GA for 24 h to obtain GA-exposed genomic DNA. After aspiration of the medium and PBS washing to remove floating cells and debris, the attached cells were harvested. The harvested cells exhibited 97.6% viability. A total of 2.5 mg of DNA was obtained from 2.26 × 10^8^ living cells. After treating this DNA sample with DNase I, phosphodiesterase I, and phosphatase, and removing the enzymes by heat denaturation, the nucleoside components were separated by reversed-phase HPLC. Complete degradation was confirmed by the analysis using a small aliquot of the reaction mixture on an analytical column (Fig. S2*C*), and then the obtained mixture was injected into the semipreparative column. Eluates around the retention times of the three standard compounds (Fig. S2*B*), as well as those containing the four canonical nucleosides detected by UV absorption, were collected separately. The collected eluates that presumably contained GA-FAPy-dG, N7-GA-Gua, and *N*^6^-GA-dA were labeled as fractions I-1, II-1, and III-1, respectively.

**Figure 3:**
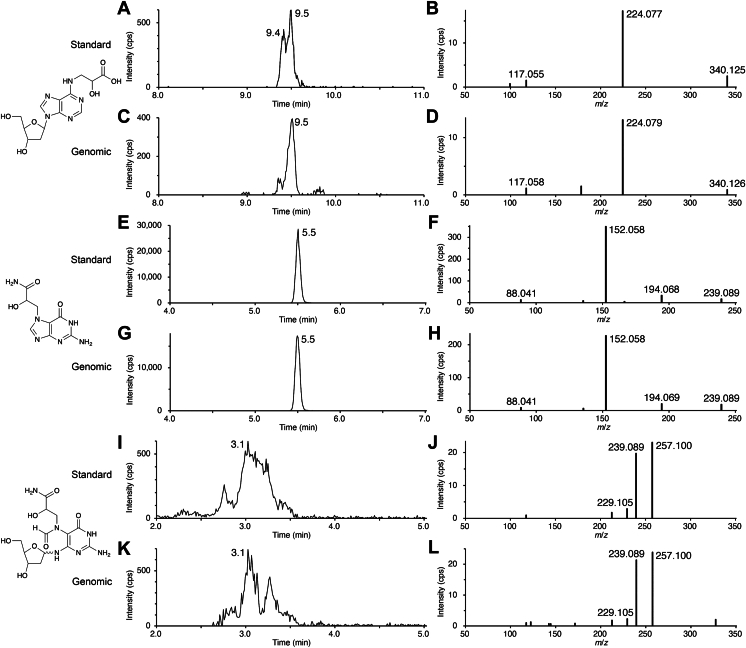
**LC**-**MS analyses of the adducts formed in GA-treated XP2OSSV cells.** Extracted ion chromatograms from TOF experiments (*left panels*) and product ion spectra acquired in the product scan experiments (*right panels*) are shown. *A* and *B*, standard *N*^6^-GA-dA (calculated *m*/*z* 340.1252), (*C* and *D*) fraction III-1, (*E* and *F*) standard N7-GA-Gua (calculated *m*/*z* 239.0887), (*G* and *H*) fraction II-1, (*I* and *J*) standard GA-FAPy-dG (calculated *m*/*z* 373.1466), and (*K* and *L*) fraction I-1. The extracted ion chromatograms were generated at *m*/*z* 340.125 (*A* and *C*), 239.089 (*E* and *G*), and 373.147 (*I* and *K*) with a mass window of 0.01 Da. The precursor ion was isolated in the mass width of 1 Da in the first mass analyzer. GA-FAPy-dG, *N*^6^-(2-deoxy-_D_-*erythro*-pentofuranosyl)-2,6-diamino-3,4-dihydro-4-oxo-5-[*N*-(2-carbamoyl-2-hydroxyethyl)formamido]pyrimidine; GA, glycidamide; *N*^6^-GA-dA, *N*^6^-(2-carboxy-2-hydroxyethyl)-2′-deoxyadenosine.

In the LC-MS analysis of fraction III-1, an extracted ion chromatogram of *m*/*z* 340.125 ([M + H]^+^ of *N*^6^-GA-dA) in the TOF experiment showed two peaks at 9.4 and 9.5 min (Fig. 3*C*). The former peak was substantially smaller than the latter, whereas the standard *N*^6^-GA-dA provided two peaks with similar intensities (Fig. 3*A*). The measured *m*/*z* values (340.126) obtained from these peaks were in good agreement with the calculated *m*/*z* value of [M + H]^+^ of *N*^6^-GA-dA (340.1252). Two product ions at *m*/*z* 117.058 and 224.079 were detected in the product ion spectra obtained in the MS/MS experiment (Fig. 3*D*). These *m*/*z* values and relative intensities were identical to those obtained for the product ions from the standard compound (Fig. 3*B*), and the *m*/*z* value of 224.079 corresponded to that of [M + H]^+^ of *N*^6^-GA-Ade (*m*/*z* 224.0778), in which the sugar moiety was lost. From the calibration curve generated with the standard compound (Fig. S4*A*), the amount of *N*^6^-GA-dA contained in this fraction was calculated to be 1.0 pmol, and the amount of dA determined by UV absorption of the collected dA peak was 2.58 μmol. These results indicated that *N*^6^-GA-Ade was formed at 39 adducts/10^8^ dA.

The detection of N7-GA-Gua and GA-FAPy-dG was also attempted. In the analysis of GA-FAPy-dG, a Waters ACQUITY UPLC Premier BEH Amide column was used, because its detection was difficult on an ordinary reversed-phase column, probably because of isomerization of the glycosidic bond and the sugar structure reported for the FAPy derivative (16). LC-MS analyses of fractions II-1 and I-1 demonstrated the presence of N7-GA-Gua and GA-FAPy-dG, whose *m*/*z* values of [M + H]^+^ were 239.090 (+1 mDa error) and 373.148 (+1 mDa error), respectively (Fig. 3, *E*–*L*). The *m*/*z* values and relative intensities of the product ions obtained in the MS/MS experiments of N7-GA-Gua and GA-FAPy-dG were in good agreement with those obtained for the product ions from the standard compounds (*right panels* of Fig. 3). From the calibration curves (Fig. S4, *B* and *C*), the amounts of N7-GA-Gua and GA-FAPy-dG were determined to be 839 and 740 pmol, respectively, and the amount of dG determined by UV absorption was 1.65 μmol. Therefore, N7-GA-Gua and GA-FAPy-dG were formed at frequencies of 5.1 × 10^4^ and 4.5 × 10^4^ adducts/10^8^ dG, respectively.

Next, we further examined whether the detection of these modified nucleotides was specific to NER-deficient cells. A normal human lung fibroblast cell line, WI-38 VA13, was also treated under the same conditions, resulting in a relative cell viability of approximately 50% compared with untreated cells (Fig. S5, *A* and *B*), similar to XP2OSSV cells (Fig. S3, *A* and *B*). The harvested cells exhibited 93.8% viability, and a total of 3.07 mg of DNA was prepared from these cells. Fractions I-2, II-2, and III-2, which were expected to contain GA-FAPy-dG, N7-GA-Gua, and *N*^6^-GA-dA, respectively, were prepared and subsequently analyzed by LC-MS in the same manner. The results obtained for *N*^6^-GA-dA (Fig. S6, *C* and *D*) and N7-GA-Gua (Fig. S6, *G* and *H*) were very similar to those from XP2OSSV cells (Fig. 3), and their formation frequencies were calculated to be 61 adducts/10^8^ dA and 4.3 × 10^4^ adducts/10^8^ dG, respectively. In the analysis of fraction I-2, the amount of GA-FAPy-dG was smaller than expected and could not be determined accurately. The lower limit of quantification (LLOQ) (signal-to-noise [S/N] ratio = 10) determined by the measurement of the GA-FAPy-dG standard was 450 pmol, but the actual amount was below this value. However, the presence of this adduct could be confirmed. In the extracted ion chromatogram obtained from the TOF measurement and the MS/MS measurement, a broad, small peak (approximately S/N = 5) was observed at a retention time of around 3.1 min (Fig. S7*E*). The protonated molecule of GA-FAPy-dG was observed at *m*/*z* 373.1473 (0.7 mDa error) in the mass spectrum obtained from the peak at 3.1 min in the extracted ion chromatogram of the TOF measurement (Fig. S7*F*). Furthermore, in the product ion spectrum, specific product ions of GA-FAPy-dG were observed at *m*/*z* 229.105, 239.089, and 257.100 (Fig. S7*H*), and the relative intensities of these ions were similar to those of the GA-FAPy-dG standard (Fig. S7*D*). Since the S/N ratio of the peak detected in fraction I-2 was a half of LLOQ, it is estimated to be approximately 225 pmol of GA-FAPy-dG, which corresponds to 1.5 × 10^4^ adducts/10^8^ dG, present in DNA.

### Preparation of oligonucleotides containing *N*^6^-GA-dA

**Figure 4:**
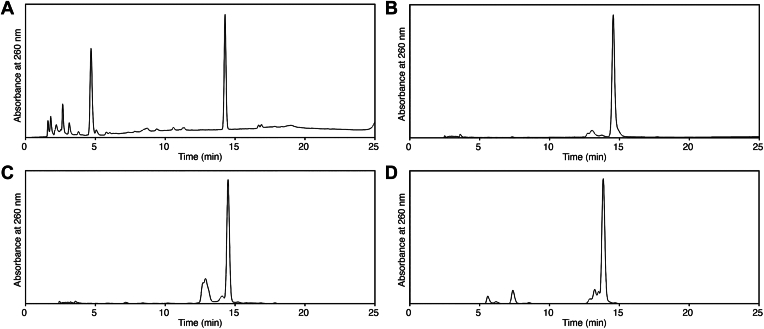
**Preparation of the 9-mer containing *N*^6^-GA-dA, p-d(TTTTXTTTT).***A*, HPLC analysis of a reaction mixture containing dA and GA in 0.1 M sodium phosphate (pH 5.0) after an incubation at 60 °C for 24 h. *B*, HPLC analysis of a reaction mixture containing the 9-mer, p-d(TTTTATTTT), and GA in pH 7.0 buffer, after an incubation at 60 °C for 24 h. The starting 9-mer had a retention time of 14.5 min. *C*, HPLC analysis of a reaction mixture containing the 9-mer and GA in pH 5.0 buffer, after incubation at 60 °C for 24 h. The product with a retention time of 13.5 min, considered to contain N1-GA-dA, was isolate. *D*, analysis of the product obtained in *C* after an incubation in pH 8.0 buffer at 60 °C for 24 h. GA, glycidamide; *N*^6^-GA-dA, *N*^6^-(2-carboxy-2-hydroxyethyl)-2′-deoxyadenosine.

In our previous study, we used a 30-mer oligonucleotide containing GA-FAPy-dG at a single position (5). Oligonucleotides containing *N*^6^-GA-dA were prepared for the present study in basically the same manner. Since the adduct formation of thymidine with GA at pH 7.0 is negligible compared with that of the other nucleosides (6), a 5′-phosphorylated 9-mer, p-d(TTTTATTTT), was treated with GA in pH 7.0 buffer at 60 °C for 24 h. However, although the adduct formation could be observed at the nucleoside level under the same conditions (Fig. 1*E*), an obvious product peak was not detected in the HPLC analysis (Fig. 4*B*). It was possible that the product, which was expected to contain *N*^6^-GA-dA, had almost the same retention time as the starting 9-mer. Therefore, we tried the reaction under the acidic conditions that yield N1-GA-dA. Since *N*^6^-GA-dA is formed even at pH 6.0 (Fig. 2*D*), a reaction in pH 5.0 buffer was tested using dA. At pH 5.0, the formation of *N*^6^-GA-dA was negligible, even when the temperature was raised to 60 °C to facilitate the reaction (Fig. 4*A*). Therefore, the 9-mer was mixed with GA in 0.1 M sodium phosphate (pH 5.0) and incubated at 60 °C for 24 h. The analysis of this mixture by reversed-phase HPLC revealed a product peak at a retention time shorter than that of the starting 9-mer (Fig. 4*C*). Its doublet-like appearance could be attributed to the stereoisomers of the GA moiety. The reaction time was prolonged to 48 h to increase the yield, and the product was isolated by HPLC purification. This product was dissolved in 0.1 M sodium phosphate (pH 8.0) and then incubated at 60 °C for 24 h. HPLC analysis revealed that the initial product was completely converted to the second product (Fig. 4*D*). After purification by HPLC, the obtained product was confirmed to be the *N*^6^-GA-dA-containing 9-mer by analysis of the nucleoside components (Fig. S8).

A 21-mer, d(GCGTACTTTTXTTTTCATGCG), in which X represents *N*^6^-GA-Ade, was prepared by ligating the adduct-containing 9-mer, a 5′-side 6-mer, d(GCGTAC), and a 3′-side 6-mer, p-d(CATGCG), using a 21-mer, d(CGCATGAAAATAAAAGTACGC), as a splint. For the preparation of a 30-mer, d(CTCGTCAGCATTTTXTTTTGACAGTCAGTG), the adduct-containing 9-mer, a 5′-side 10-mer, d(CTCGTCAGCA), a 3′-side 11-mer, p-d(GACAGTCAGTG), and a splint 21-mer, d(ACTGTCAAAATAAAATGCTGA), were used. After incubation at 16 °C for 20 h, the reaction mixtures were analyzed by reversed-phase HPLC under heat-denaturing conditions (Fig. S9), and the products purified by HPLC were analyzed by mass spectrometry (Fig. S10).

### Analysis of duplex stabilities to determine base-pair formation

To analyze the base-pair formation of *N*^6^-GA-dA, we performed thermodynamic studies. The 21-mers, d(GCGTACTTTTXTTTTCATGCG), with or without the GA adduct at X, were hybridized to 13-mer oligonucleotides, d(TGAAAAYAAAAGT), in which Y represents A, G, C, or T. Shorter complementary oligonucleotides were chosen to maximize the effect of the X·Y base pair, whereas relatively long flanking sequences at the 5′ and 3′ ends were required for the preparation of the adduct-containing strand using DNA ligase. Thermal melting curves were monitored at 260 nm at total oligonucleotide concentrations (*C*_t_) ranging from 4.0 to 20.0 μM. The thermodynamic parameters for each duplex were determined from the *T*_m_ data by the van’t Hoff method, assuming a two-state model for duplex melting (17). The 1/*T*_m_ *versus* ln(*C*_t_/4) plots are shown in Fig. S11, and the obtained parameters together with the *T*_m_ values at *C*_t_ = 20.0 μM are listed in Table 1. Although the adduct formation decreased the thermodynamic stability of the duplex, the duplex was most stable when thymine was the opposite base, regardless of the presence or absence of the adducted GA moiety. These results strongly suggest that *N*^6^-GA-dA can form a base pair with thymidine in the same manner as dA.

**Table 1:** Thermodynamic parameters for duplex formation of d(GCGTACTTTTXTTTTCATGCG) · d(TGAAAAYAAAAGT)

X · Y	*T*_m_^a^	Δ*H*°	Δ*S*°	Δ*G*°^b^	ΔΔ*G*°^b^^,^^c^
	(°C)	(kcal mol^−^^1^)	(cal mol^−^^1^ K^−^^1^)	(kcal mol^−^^1^)	(kcal mol^−^^1^)
A · A	24.4	−82.2 ± 1.8	−252 ± 6	−7.09 ± 0.01	
A · G	30.5	−61.7 ± 6.8	−179 ± 22	−8.26 ± 0.07	
A · C	26.9	−69.1 ± 4.0	−206 ± 14	−7.62 ± 0.02	
A · T	39.5	−74.8 ± 9.2	−216 ± 30	−10.57 ± 0.32	
*N*^6^-GA-Ade · A	18.5	−74.2 ± 6.7	−231 ± 23	−5.51 ± 0.20	1.58 ± 0.21
*N*^6^-GA-Ade · G	20.9	−67.3 ± 3.9	−205 ± 13	−6.25 ± 0.08	2.01 ± 0.15
*N*^6^-GA-Ade · C	18.4	−74.1 ± 4.5	−230 ± 16	−5.51 ± 0.13	2.11 ± 0.15
*N*^6^-GA-Ade · T	30.5	−89.5 ± 8.4	−271 ± 28	−8.77 ± 0.09	1.80 ± 0.41

a *C*_t_ = 20.0 μM.

b At 25 °C.

c Δ*G*°(*N*^6^-GA-Ade·Y) − Δ*G*°(A·Y).

### Incorporation of nucleotides opposite *N*^6^-GA-dA

**Figure 5:**
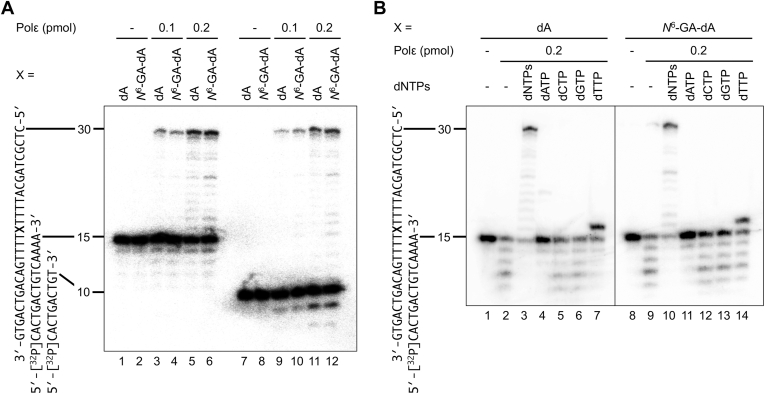
**Effects of *N*^6^-GA-dA on DNA synthesis.***A*, primer extension assays using 30-mer oligonucleotides, d(CTCGTCAGCATTTTXTTTTGACAGTCAGTG), where X represents dA or *N*^6^-GA-dA, as templates. The [^32^P]-labeled complementary 15-mer and 10-mer primers, d(CACTGACTGTCAAAA) and d(CACTGACTGT), respectively, were annealed to the 30-mer templates. The primer-template substrates were incubated with or without the catalytic fragment of human Polε at 37 °C for 15 min. The reaction mixtures were subjected to denaturing PAGE and visualized by autoradiography. *B*, single-nucleotide incorporation assay. The [^32^P]-labeled 15-mer primer-templates were incubated with the catalytic fragment of human Polε in the presence of all four deoxynucleoside triphosphates (dNTPs) or individual dATP, dCTP, dGTP, or dTTP at 37 °C for 15 min. The reaction mixtures were subjected to denaturing PAGE and visualized by autoradiography. GA, glycidamide; *N*^6^-GA-dA, *N*^6^-(2-carboxy-2-hydroxyethyl)-2′-deoxyadenosine.

To examine the effects of *N*^6^-GA-dA in the template strand on DNA synthesis, we performed primer extension assays using the 30-mer oligonucleotide containing *N*^6^-GA-dA hybridized to a complementary 15-mer primer (Table S1), placing the adduct immediately after the primer terminus. Human DNA polymerase ε (Polε), a replicative DNA polymerase responsible for normal DNA replication, was able to bypass *N*^6^-GA-dA with an efficiency comparable to that of unmodified dA (Fig. 5*A*). However, when *N*^6^-GA-dA was present in the template, a slight accumulation of a 22-mer intermediate product was observed compared with the dA template (Fig. 5*A*, lanes 5 and 6). Using a 10-mer primer also produced minor accumulations of 15- to 17-mer products, together with the 22-mer intermediate (Fig. 5*A*, lanes 11 and 12). Time-course experiments further revealed that the presence of *N*^6^-GA-dA slightly reduced primer extension efficiency at shorter reaction times. However, the reaction eventually reached the fully extended 30-mer product (Fig. S12). In a single nucleotide incorporation assay, dTTP was exclusively incorporated opposite *N*^6^-GA-dA (Fig. 5*B*). When the template contained normal A at position 16 and dATP was supplemented as a deoxynucleotide substrate (Fig. 5*B*, lane 4), a faint band was observed at position 20. This can be explained by the presence of four consecutive T residues (positions 17–20) following X (position 16). If dATP was occasionally misincorporated opposite A at position 16, DNA synthesis could proceed along the T stretch, resulting in the band observed at position 20. Since truncated primers appeared because of the inherent proofreading exonuclease activity of Polε, we repeated the assay using the Klenow fragment for clarity and obtained results consistent with those generated with Polε without the truncated primer bands (Fig. S13).

### Effects of *N*^6^-GA-dA on DNA replication efficiency and mutagenicity in human cells

**Figure 6:**
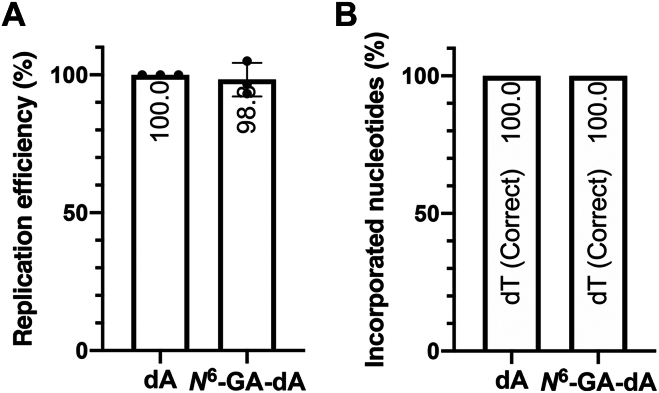
**Intracellular TLS assay of *N*^6^-GA-dA.***A*, replication efficiency of *N*^6^-GA-dA, normalized to that of the concurrent control plasmid carrying dA at the corresponding site. Replication efficiency was calculated as the ratio of the modified strand progeny (SmiI-resistant clones) to total clones analyzed. Data represent the mean ± standard deviation of three independent experiments. No statistically significant changes were detected by a two-tailed unpaired *t* test with Welch’s correction. *B*, nucleotides incorporated opposite the adduct site in the modified strand progeny. SmiI-resistant PCR products were analyzed by Sanger sequencing. *N*^6^-GA-dA, *N*^6^-(2-carboxy-2-hydroxyethyl)-2′-deoxyadenosine; TLS, translesion synthesis.

To investigate the mutagenicity of *N*^6^-GA-dA in human cells, we introduced the *N*^6^-GA-dA oligonucleotide into the shuttle vector used in our previous study (5). XP4PASV cells were transfected with the shuttle vector carrying a single dA or *N*^6^-GA-dA at a specific position, allowing replication by the cellular replication machinery. The replication efficiency, determined by the ratio of the adduct-carrying strand to its complementary undamaged strand, was comparable to that of the control vector carrying unmodified dA at the corresponding position (Fig. 6*A*). Moreover, no mutations were detected at or near the adduct site among hundreds of progeny of the *N*^6^-GA-dA vector (Table 2 and Fig. 6*B*), indicating that the upper 95% confidence limit of mutation frequency was below 1.04%. These results suggest that *N*^6^-GA-dA is neither a DNA replication-blocking lesion nor a mutagenic lesion.

**Table 2:** Sequencing analysis of the modified strand progeny from the intracellular site-specific translesion synthesis assay

Modified strand progeny	dA	*N*^6^-GA-dA
Correct		
5′-TGTCAAAATAAAATGCTGACGAG-3′	259 (100%)	288 (100%)
Incorrect	0 (0%)	0 (0%)
Total	259 (100%)	288 (100%)

The complementary strand of the modified strand was sequenced. The adduct site is underlined.

## Discussion

In studies on the carcinogenicity of AA, GA-adducted bases, N7-GA-Gua and N3-GA-Ade, which are released from DNA strands after adduct formation, have been recognized as important biomarkers (18). However, we recently demonstrated that GA-FAPy-dG, which remains in DNA, induces G:C > A:T transitions (5). The formation of this damaged nucleoside was only shown in the reaction of dG with GA in our previous study, but in the present study, the presence of GA-FAPy-dG was confirmed through detection in DNA isolated from GA-treated cells. In the case of adenine as well, the adducted base remaining in DNA is important for understanding the mutagenic potential of AA. Regarding the reaction of GA with dA, previous studies revealed the formation of N1-GA-dA and its conversion to *N*^6^-GA-dA by the Dimroth rearrangement (7, 8), which has also been reported for 1-methyladenosine (19) and N1 adducts of dA with other oxacyclopropane-containing compounds (20, 21, 22, 23). In the Dimroth rearrangement, the first reaction is the attack of a hydroxide ion at C2 of adenine, followed by the cleavage of the N1-C2 bond (19), and highly alkaline conditions are reportedly required for efficient rearrangement to proceed (24). We first confirmed that these two products were obtained from the reaction between dA and GA at pH 7.0, and the products obtained predominantly in pH 6.0 and 8.0 solutions at 37 °C were N1-GA-dA and *N*^6^-GA-dA, respectively (Fig. 1). Since the complete conversion of N1-GA-dA to *N*^6^-GA-dA at 60 °C in a pH 7.0 solution (Fig. 1*E*) was an unexpected result, we analyzed the pH dependency of this reaction. As shown in Figure 2*D*, the Dimroth rearrangement proceeded at a substantially high rate in a pH 7.0 solution at 37 °C. Therefore, we focused on *N*^6^-GA-dA as the stable adenine adduct present in DNA.

To ensure the biological significance of studying *N*^6^-GA-dA, we tried to detect this adduct in cellular DNA. Genomic DNA was isolated from GA-treated XP2OSSV cells, and after degradation to its nucleoside components, a fraction putatively containing *N*^6^-GA-dA was obtained by HPLC separation. An LC-MS analysis revealed the existence of *N*^6^-GA-dA in this fraction (Fig. 3, *A*–*D*), and its amount was calculated to be 39 adducts/10^8^ nucleotides following treatment with 1 mM GA for 24 h, a condition optimized for recovering GA-treated genomic DNA from living cells.

In parallel, we tried to detect and quantify two types of guanine adducts, N7-GA-Gua and GA-FAPy-dG, which we investigated in our previous study (5). These two adducts were also present (Fig. 3, *E*–*L*), and their amounts were 5.1 × 10^4^ and 4.5 × 10^4^ adducts/10^8^ nucleotides, respectively. It is possible that the amount of N7-GA-Gua was underestimated, because this compound that cleaved from DNA in cells was excluded from the LC-MS sample, as confirmed by the experiments shown in Fig. S14. The detected N7-GA-Gua molecules were presumably generated during the enzymatic degradation of the isolated DNA and the enzyme heat-denaturation step. Nevertheless, the level of N7-GA-Gua formation is very similar to the reported value of 4.9 × 10^4^ adducts/10^8^ nucleotides (11). Based on these results, it is plausible that a large amount of GA-FAPy-dG forms in cellular genomic DNA, compared with our previous estimation based on the reaction of GA with dG (5). The discrepancy between GA-FAPy-dG formation in genomic DNA and in monomeric dG might be attributed to the reduction of the depurination rate constant of N7-alkyl-dG *via* interactions with histone tails (25). While the amount of GA-FAPy-dG was successfully determined in the genomic DNA of NER-deficient XP2OSSV cells, the S/N ratio of GA-FAPy-dG peak derived from NER-proficient WI-38 VA13 cells was approximately 5, falling below the LLOQ (S/N = 10). Nevertheless, the measured *m/z* of [M + H]^+^ of GA-FAPy-dG and MS/MS analysis provided sufficient evidence to confirm the presence of GA-FAPy-dG in WI-38 VA13 cells. Although the level of GA-FAPy-dG was below the LLOQ, it was estimated to be on the order of 10^4^ GA-FAPy-dG/10^8^ nucleosides. DNA adduct levels ranging from 10^2^ to 10^4^/10^8^ nucleotides induced by various carcinogens *in vivo* contribute to a 50% tumor incidence (26), suggesting that GA-FAPy-dG is relatively abundant even in NER-proficient cells. Our previous study demonstrated that GA-FAPy-dG exhibits marked mutagenicity (exceeding 10% in human cells) (5), indicating that its detection at this level is biologically relevant. By contrast, the levels of replication-unblocking *N*^6^-GA-dA and of N7-GA-Gua, which reflect the presence of its precursor N7-GA-dG, were comparable regardless of NER status. The observation that GA-FAPy-dG, which undergoes structural alteration through imidazolium ring opening, can be subject to NER is consistent with the damage verification mechanism involving the steric gate of XPD (27).

Compared with the production of these guanine adducts, much less *N*^6^-GA-dA is formed. Another finding in the LC-MS analysis was that the peak of one of the two stereoisomers of *N*^6^-GA-dA was very small (Fig. 3*C*), whereas its standard compound gave two peaks (Fig. 3*A*). A possible reason for this difference is that one of the stereoisomers was lost during the HPLC fractionation step because the peaks could not be monitored by UV absorption. Alternatively, one of the stereoisomers of GA may have been unable to react with adenine in the double-stranded DNA structure because of steric hindrance.

Oligonucleotides containing *N*^6^-GA-dA were prepared successfully by using a short sequence surrounded by thymine, which does not react with GA at pH values lower than 7 (6). The *N*^6^-GA-dA-containing product was not separated from the parent 9-mer, p-d(TTTTATTTT), when the pH 7.0 mixture was incubated at 60 °C for 24 h. Therefore, the N1-GA-dA-containing 9-mer was prepared at pH 5.0 first, and then the adduct was converted to *N*^6^-GA-dA by subsequent incubation at pH 8.0. Using DNA ligase in the presence of a splint oligonucleotide, sequence-defined 21- and 30-mers were prepared, and the 21-mer was used for the thermodynamic analysis of base-pair formation. The results revealed that *N*^6^-GA-Ade can form a base pair with thymine in the same manner as adenine (Table 1). This finding can be explained by the chemical structure of *N*^6^-GA-Ade. A hydrogen atom that can participate in base-pair formation with thymine exists at the nitrogen attached to C6. This type of base pairing was previously shown for the *N*^6^-methyladenine·uracil pair in an NMR study of an RNA duplex (28). In the present study, the duplexes uniformly became unstable by the adduct formation, as shown in the ΔΔ*G*° column in Table 1. Similar destabilization has been reported for the *N*^6^-methyladenine-containing RNA duplexes, where the effect of the *N*^6^-methyl group was attributed to the high-energy anticonformation of the methylamino group (28). The same reasoning applies to our duplexes containing *N*^6^-GA-Ade. As shown in Table 1, the larger negative value of Δ*S*° obtained for the *N*^6^-GA-Ade·T pair than that for the A·T pair contributed to the duplex destabilization by the adduct formation (1.8 kcal/mol at 25 °C), and a similar tendency was reported for duplexes containing *N*^6^-methyladenosine (29). This observation can be explained by the reason that fixation of the methylamino conformation as *anti* is entropically unfavorable. The electrostatic repulsion between the carboxy group, produced by hydrolysis of the adducted GA, and the phosphate backbone may provide an extra destabilization effect in the duplex.

In the primer extension assay, *N*^6^-GA-dA did not block DNA synthesis by the human replicative DNA polymerase Polε. Replicative DNA polymerases have highly stringent catalytic centers that ensure the strict nucleotide incorporation accuracy at the expense of being less accommodating to modified nucleotides in templates. Despite this property, Polε efficiently bypassed *N*^6^-GA-dA at a level comparable to unmodified dA (Fig. 5*A*), indicating that *N*^6^-GA-dA does not pose a strong obstacle to DNA polymerases. However, the slight accumulation of intermediate products, particularly at shorter time points (Fig. S12), suggested that the processivity of Polε was reduced on the *N*^6^-GA-dA template. The decreased thermodynamic stability of the duplex containing *N*^6^-GA-dA may contribute to the reduction in processivity. Nonetheless, the reaction eventually yielded the fully extended 30-mer product at 15 min. The discrepancy that the DNA duplex is thermodynamically destabilized yet high-fidelity polymerases bypass the lesion can be explained by differences in the experimental context. In the thermodynamic assay, the adduct was located in the middle of the duplex, and the duplex stability solely reflected the intrinsic properties of the DNA helix. In contrast, in the primer extension assay, the adduct was positioned at the primer terminus, and the reaction occurred within the enzyme’s active site. In addition to duplex stability, incorporation efficiency depends on base pairing between the adduct and the incoming nucleotide as well as electrostatic interactions within the polymerase active site. The intracellular replication efficiency of the modified strand carrying a single *N*^6^-GA-dA was comparable to that of the unmodified strand carrying canonical dA (Fig. 6*A*). The reduced efficiency of DNA synthesis observed in the primer extension assay, but not in the overall replication efficiency in cells, might be attributed to the fact that only the catalytic fragment of Polε was used in the primer extension assay, whereas in cells, the Polε holoenzyme functions in coordination with other replication factors such as PCNA. Taken together, the lack of appreciable stalling by Polε and replication efficiency in cells indicate that *N*^6^-GA-dA does not act as a DNA replication-blocking lesion.

The decrease in the thermodynamic stability of the duplex occurs regardless of the opposite base. This means that although base pairing between *N*^6^-GA-Ade and T is destabilized compared with unmodified A, similar destabilization also occurs with A, G, or C, as indicated by the comparable ΔΔ*G*° values. Consequently, it does not affect the base selection at the nucleotide incorporation step. Consistently, *N*^6^-GA-dA did not exhibit miscoding potential in single nucleotide incorporation assays, where dTTP was exclusively incorporated opposite *N*^6^-GA-dA by both Polε and Klenow fragments (Figs. 5*B* and S13). Moreover, the intracellular translesion synthesis assay did not detect any mutations induced by *N*^6^-GA-dA in human cells (Table 2 and Fig. 6*B*). Collectively, our physicochemical, biochemical, and cell-based studies suggest that *N*^6^-GA-dA is not a mutagenic lesion.

The formation of stable (nondepurinating) DNA adducts, other than N7-GA-Gua and N3-GA-Ade, has been detected in reactions of GA with nucleosides (7, 8). However, these adducts have received little attention in toxicological evaluations of AA, likely because they have not been detected *in vivo*. The T:A > C:G transition in the GA signature is considered to correspond to N1-GA-dA (11, 12) because the N1 position of dA is involved in base pairing, although no direct evidence has been provided. While N1-GA-dA undergoes the Dimroth rearrangement to *N*^6^-GA-dA, this reaction typically occurs under high pH conditions, and the potential presence of *N*^6^-GA-dA in biological samples has not been addressed. In this study, we found that at least two stable GA adducts, *N*^6^-GA-dA and GA-FAPy-dG, were actually formed in cellular DNA following exposure to GA, providing new insights into the molecular mechanisms underlying AA/GA-induced mutagenesis. While previous studies have shown that certain *N*^6^-dA adducts, such as *R*- and *S*-styrene oxide adducts and (*R*,*R*)- and (*S*,*S*)-diolepoxide adducts, induce replication inhibition and/or base substitution mutations (30, 31), our results indicate that *N*^6^-GA-dA causes neither detectable replication blockage nor mutagenesis. However, highly mutagenic GA-FAPy-dG was present in much greater amounts than *N*^6^-GA-dA, and its level was comparable to that of N7-GA-Gua. These findings strongly emphasize the importance of analyzing the mutational potential of chemically stable GA adducts existing in DNA.

## Experimental procedures

### General methods

Nucleosides, acetonitrile, and salts for buffer preparation were purchased from FUJIFILM Wako Pure Chemical Corp. GA and the 2 M solution of triethylammonium acetate (TEAA) were purchased from Merck Sigma-Aldrich and Glen Research, respectively. Oligonucleotides were synthesized at Tsukuba Oligo Service Co Ltd and purified by HPLC at Osaka University. HPLC analyses were performed on a Gilson gradient-type analytical system equipped with a Waters 2998 photodiode array detector and a Shimadzu CTO-20A column oven. For purification, elution was monitored by a Gilson 151 or Hitachi 1410 UV detector. LC-MS analyses were performed on a SCIEX X500R quadrupole TOF system coupled with a Waters ACQUITY UPLC H-class system. Melting curves of the oligonucleotide duplexes were measured on a JASCO V-730 spectrophotometer equipped with a PAC-743 water-cooled Peltier cell holder.

### Adduct formation of GA with dA

Solutions containing dA (15 nmol) and GA (7.5 μmol) in 50 mM sodium phosphate (30 μl) were incubated at 37 °C for 72 h or at 60 °C for 24 h. Aliquots were analyzed by HPLC using an Xbridge C18 5 μm column (4.6 × 150 mm; Waters Corp) at a flow rate of 1.0 ml/min with a gradient of 0% to 7.5% acetonitrile (20 min) in 0.1 M TEAA (Fig. 1, *B*–*E*).

### Rearrangement of N1-GA-dA to *N*^6^-GA-dA

N1-GA-dA was prepared by incubating a solution containing dA (50 nmol) and GA (25 μmol) in 0.1 M sodium phosphate (pH 5.0, 50 μl) at 60 °C for 24 h and purified by HPLC using an Xbridge C18 5 μm column (4.6 × 150 mm) at a flow rate of 1.0 ml/min with a gradient of 0% to 7.5% acetonitrile (20 min) in 10 mM ammonium acetate. The eluate was evaporated to dryness and desalted by coevaporation with water, and the yield was determined by UV absorption using the molecular extinction coefficient reported for 1-methyl-dA (32). This product, N1-GA-dA (1.0 nmol), was dissolved in 0.1 M sodium phosphate (pH 7.0 or pH 8.0, 50 μl), and after an incubation at 37 °C (pH 7.0) or 60 °C (pH 8.0) for 24 h, aliquots were analyzed by HPLC, using an Xbridge C18 5 μm column (4.6 × 150 mm) at a flow rate of 1.0 ml/min with a gradient of 0% to 7.5% acetonitrile (20 min) in 0.1 M TEAA (Fig. 2, *A*–*C*). Time-course experiments were performed by HPLC analyses of reaction mixtures containing dA and GA in pH 6.0, 7.0, and 8.0 buffers after incubations for 6, 12, 24, 36, and 48 h. The yields of *N*^6^-GA-dA were calculated from the peak areas monitored at the absorption maxima of N1-GA-dA and *N*^6^-GA-dA, using the molecular extinction coefficients reported for 1-methyl-dA and *N*^6^-methyl-dA (32).

### Preparation of the GA adduct standards for LC-MS analysis

GA-FAPy-dG and N7-GA-Gua were prepared by incubating a solution (100 μl) containing dG (100 nmol) and GA (50 μmol) in 0.1 M sodium phosphate (pH 7.0) at 37 °C for 48 h (5). For *N*^6^-GA-dA, 1.0 M GA (25 μl) was added to a solution of dA (50 nmol) in 0.2 M sodium phosphate (pH 7.0, 25 μl), and the mixture was incubated at 60 °C for 24 h. The three products were purified by HPLC using an Xbridge C18 5 μm column (4.6 × 150 mm) at a flow rate of 1.0 ml/min with a gradient of 0% to 7.5% acetonitrile (20 min) in 10 mM ammonium acetate. The eluates were evaporated to dryness and desalted by coevaporation with water. The purified compounds were analyzed under the same conditions (Fig. S1). The yields of the obtained products were determined by UV absorption, using the molecular extinction coefficients reported for the *N*-methyl adducts (33, 34, 35).

### Cell lines and cultures

SV40-transformed human skin fibroblasts XP2OSSV and XP4PASV were derived from xeroderma pigmentosum group A and group C patients, respectively. These cell lines, as well as an SV40-transformed human normal lung fibroblast line, WI-38 VA13, were cultured in Dulbecco’s modified Eagle’s medium (Nissui Pharmaceutical) supplemented with 10% fetal bovine serum (Sigma; lot no.: 14B247) at 37 °C in 5% CO_2_.

### Sample preparation for GA adduct detection in genomic DNA by LC-MS

To determine the optimal conditions for GA treatment, XP2OSSV cells were seeded in a 24-well plate and incubated at 37 °C in 5% CO_2_. After overnight culture, cells were treated with 0.1 to 1.0 mM GA for 24 and 48 h. Cell morphology was monitored by phase-contrast microscopy, and viability was measured at the end of treatment using CellTiter 96 Aqueous One Solution (Promega). Based on the results (Fig. S3), XP2OSSV cells were seeded in 15 × 15-cm dishes and treated with 1.0 mM GA at 37 °C for 24 h in 5% CO_2_. After treatment, the medium was aspirated, cells were rinsed with PBS, detached using TrypLE Express (Thermo Fisher Scientific), and rinsed with ice-cold PBS. Cell viability and number of living cells were determined to be 97.6% and 2.26 × 10^8^, respectively, using the trypan blue dye exclusion test. Genomic DNA was isolated with a QIAamp DNA Blood Maxi Kit (Qiagen). Briefly, the cells were divided into three tubes and resuspended in 10 ml PBS per tube. After adding 500 μl of Qiagen Protease, the cells were lysed with Buffer AL (12 ml), incubated at 70 °C for 10 min, mixed with ethanol (10 ml), and then applied to a QIAamp Maxi column for genomic DNA purification. A total of 2.5 mg in 5.0 ml of genomic DNA was obtained from XP2OSSV cells. For WI-38 VA13 cells, sample preparation was performed under the same treatment conditions in 20 × 15-cm dishes, yielding 8.16 × 10^8^ living cells with 93.8% viability as determined by the trypan blue dye exclusion test. The cells were divided into eight tubes and subjected to genomic DNA preparation using the same procedure, yielding a total of 3.07 mg in 16.9 ml of genomic DNA. The purified genomic DNA was diluted with water (17 ml), and to this solution, RQ1 DNase 10X Reaction Buffer (Promega; 2.5 ml) and RQ1 RNase-Free DNase (Promega; 0.5 ml, 500 units) were added. After an incubation at 37 °C for 2 h, 0.5 M Tris-HCl (pH 7.0, 3.0 ml), Antarctic Phosphatase (New England Biolabs; 0.5 ml, 2500 units), and phosphodiesterase I from *Crotalus adamanteus* (Worthington Biochemical Corp; 1.0 ml, 20 units) were added. This mixture was incubated at 37 °C for 4.5 h, and after the addition of 0.2 M sodium phosphate (pH 6.0, 5.0 ml), the enzymes were denatured by heating the solution at 95 °C for 10 min. The resulting solution was concentrated by rotary evaporation to ca. 3 ml, and insoluble materials were removed by centrifugation at 12,000 rpm for 2 min. The supernatant was further concentrated on a SpeedVac concentrator (Thermo Scientific Savant) and then centrifuged, yielding ca. 1.8 ml of solution. A small aliquot of this solution (10 μl) was analyzed by HPLC using an XBridge C18 5 μm column (4.6 × 150 mm) at a flow rate of 1.0 ml/min with a gradient of 0% to 7.5% acetonitrile (20 min) in 10 mM ammonium acetate (Fig. S2*C*). The solution of degradation products (0.9 ml at one time) was injected into an XBridge Prep C18 5 μm column (10 × 250 mm; Waters Corp) equilibrated with 10 mM ammonium acetate, and elution was performed with a gradient of 0% to 7.5% acetonitrile (40 min) in 10 mM ammonium acetate at a flow rate of 2.0 ml/min. As determined by the analysis using the unmodified nucleosides and the standard compounds (Fig. S2, *A* and *B*), the 14.5 to 17.5-min, 19.5 to 22.5-min, and 30.0 to 33.0-min fractions, which presumably contained GA-FAPy-dG, N7-GA-Gua, and *N*^6^-GA-dA, respectively, were collected and labeled as fractions I-1, II-1, and III-1 (XP2OSSV cells) and fractions I-2, II-2, and III-2 (WI-38 VA13 cells). These fractions were evaporated to dryness and desalted by coevaporation with water. The peaks of the canonical nucleosides detected by UV absorption were also collected and treated in the same manner.

### LC-MS analysis

Fraction III was dissolved in 10 mM ammonium acetate (30 μl), and an aliquot (3.0 μl) was analyzed on an InertSustain AQ-C18 PEEK column (1.9 μm, 2.1 × 150 mm; GL Sciences, Inc) at a flow rate of 0.2 ml/min, using a gradient of 3.8% to 9.5% acetonitrile (15 min) in 10 mM ammonium acetate. After analyzing the samples obtained from XP2OSSV, the retention capacity of the InertSustain AQ-C18 PEEK column decreased, so a new column was used for the analysis of WI-38 VA13. The retention time varied depending on the column, but the resolution between the nucleoside and the adjacent GA adduct was sufficient for separation (resolution >3.0) for both columns. The mass spectrometer was operated in the positive electrospray ionization mode with a capillary voltage of 5500 V. Data were acquired using the TOF mode to obtain extracted ion chromatograms with high mass resolution for quantification, and the accurate mass of *N*^6^-GA-dA was determined from the peak observed in the extracted ion chromatograms of *m*/*z* 340.125 ± 0.005 Da. For quantification, a calibration curve was obtained from the averages of the peak areas (n = 3), using 20, 40, 60, 80, and 100 nM *N*^6^-GA-dA solutions in 10 mM ammonium acetate (Fig. S4*A*). In addition, MS/MS measurements were performed to ensure the identification of *N*^6^-GA-dA. The precursor ion for the MS/MS experiment (product ion scan) was set to [M + H]^+^ at *m*/*z* 340.1 in the mass width of 1 Da, and the collision energy was 20 V. The analyses of fraction II (N7-GA-Gua) were performed in a similar manner, except that the calibration curve was obtained between 0.1 and 1.0 μM (Fig. S4*B*), and the collision energy was 25 V. For fraction I (GA-FAPy-dG), an ACQUITY UPLC Premier BEH Amide column (1.7 μm, 2.1 × 150 mm; Waters Corp) was used at a flow rate of 0.3 ml/min with a gradient of 80% to 50% eluent B (5 min) in eluent A (A, water containing 0.1% formic acid and 10 mM ammonium formate; B, 9:1 mixture of acetonitrile and water containing 0.1% formic acid and 5 mM ammonium formate), and the calibration curve was between 5.0 and 20 μM (Fig. S4*C*). The injection volume was 5 μl for the analysis of XP2OSSV. For the WI-38 VA13 sample, 10 μl was injected because of a decrease in the sensitivity caused by contamination of the mass spectrometer. The collision energy for MS/MS was set to 18 V.

### Confirmation of N7-GA-Gua loss during DNA purification

A QIAGEN DNeasy Blood & Tissue Kit was used according to the manufacturer’s protocol. In brief, N7-GA-Gua (5 nmol) dissolved in Buffer AL (200 μl) or the buffer alone was mixed with ethanol (200 μl) and loaded onto a DNeasy Mini Spin Column. After centrifugation, the membrane was washed with buffers AW1 and AW2 (500 μl each) in this order, and the retained compound was eluted with buffer AE (200 μl). An aliquot of the eluate was analyzed by HPLC, using an XBridge C18 5 μm column (4.6 × 150 mm) at a flow rate of 1.0 ml/min with a gradient of 0% to 2.5% acetonitrile (20 min) in 10 mM ammonium acetate. The N7-GA-Gua standard and an eluate without N7-GA-Gua were analyzed under the same conditions (Fig. S14).

### Preparation of a 9-mer containing *N*^6^-GA-dA

An aqueous solution of a 5′-phosphorylated 9-mer, p-d(TTTTATTTT), (350 nmol in 700 μl) was mixed with 0.2 M sodium phosphate (pH 5.0, 875 μl), and a 1.0 M solution of GA (175 μl) was added. This mixture was incubated at 60 °C for 48 h, and a small aliquot (4 μl) was analyzed by HPLC, using an Inertsil ODS-3 5 μm column (4.6 × 250 mm; GL Sciences, Inc) at a flow rate of 1.0 ml/min with a gradient of 9% to 15% acetonitrile (20 min) in 0.1 M TEAA, in a column oven set at 50 °C. The product was purified by HPLC under the same conditions. The eluate was concentrated by rotary evaporation, and TEAA was removed by coevaporation with water. The residue was dissolved in 0.1 M sodium phosphate (pH 8.0), and this solution was incubated at 60 °C for 24 h. The product was analyzed and purified by HPLC under the above-mentioned conditions. The purity of the final product was confirmed by anion-exchange HPLC, using a TSKgel DEAE-2SW column (4.6 × 250 mm; Tosoh Corp) at a flow rate of 1.0 ml/min with a gradient of 0.4 to 0.8 M ammonium formate (20 min) in 20% acetonitrile, in a column oven set at 50 °C. The presence of *N*^6^-GA-dA in the obtained oligonucleotide was confirmed by nucleoside analysis. A solution of the 9-mer (1.5 nmol in 44 μl) was mixed with S1 Nuclease 10X Reaction Buffer (Promega; 5.0 μl) and S1 Nuclease (Promega; 1.0 μl, 79 units). After an incubation at 37 °C for 1 h, 0.5 M Tris-HCl (pH 7.0, 6.0 μl), Antarctic Phosphatase (New England Biolabs, 1.0 μl, 5 units), and phosphodiesterase I from *C. adamanteus* (Worthington Biochemical Corp; 2.0 μl, 0.04 unit) were added, and the mixture was further incubated at 37 °C for 3 h. The products were analyzed by HPLC, using an XBridge C18 5 μm column (4.6 × 150 mm) at a flow rate of 1.0 ml/min with a gradient of 0% to 7.5% acetonitrile (20 min) in 0.1 M TEAA (Fig. S8).

### Preparation of 21-mer and 30-mer oligonucleotides containing *N*^6^-GA-dA

For the preparation of the 21-mer, the 9-mer, p-d(TTTTXTTTT) in which X represents *N*^6^-GA-Ade (the base moiety of *N*^6^-GA-dA), (20 nmol) was mixed with d(GCGTAC) (24 nmol), p-d(CATGCG) (24 nmol), and d(CGCATGAAAATAAAAGTACGC) (24 nmol) in water (860 μl). This solution was heated at 50 °C for 5 min and cooled gradually to 16 °C. After the addition of T4 DNA Ligase Reaction Buffer (New England Biolabs; 100 μl) and T4 DNA Ligase (New England Biolabs; 40 μl, 16,000 units), the mixture was incubated at 16 °C for 20 h. The product was analyzed and purified by HPLC, using an Inertsil ODS-3 5 μm column (4.6 × 250 mm) at a flow rate of 1.0 ml/min with a gradient of 9% to 13% acetonitrile (20 min) in 0.1 M TEAA, in a column oven set at 60 °C (Fig. S9*A*). For the 30-mer, d(CTCGTCAGCA), p-d(TTTTXTTTT), p-d(GACAGTCAGTG), and d(ACTGTCAAAATAAAATGCTGA) were used, and the acetonitrile gradient was 9% to 15% (Fig. S9*B*). The oligonucleotides were analyzed by mass spectrometry at Hokkaido System Science Co, Ltd, using electrospray ionization in the negative ion mode (Fig. S10).

### Measurement of melting curves

Two oligonucleotides, d(GCGTACTTTTXTTTTCATGCG) and d(TGAAAAYAAAAGT), in which X and Y represent A or *N*^6^-GA-Ade and A, G, C, or T, respectively (2.0 nmol each) were dissolved in water (100 μl), and the solutions were heated at 65 °C for 3 min and cooled gradually to room temperature. Melting curves were measured at total oligonucleotide concentrations (*C*_t_) of 4.00, 5.94, 8.96, 13.4, and 20.0 μM, in 10 mM sodium phosphate (pH 7.0) containing 100 mM NaCl, in the temperature range from 10 °C to 60 °C. *T*_m_ values were obtained by the derivative method, using the data processing software supplied by the manufacturer (JASCO). These experiments were performed in triplicate. The reciprocal of *T*_m_ was plotted against ln*C*_t_/4 (Fig. S11), and the thermodynamic parameters were obtained from the equations 1/*T*_m_ = (*R*/Δ*H*°)ln*C*_t_/4 + Δ*S*°/Δ*H*° and Δ*G*° = Δ*H*° − *T*Δ*S*°, in which *R* is the gas constant and *T* is the absolute temperature.

### Primer extension assay

The 30-mer template oligonucleotides, d(CTCGTCAGCATTTTXTTTTGACAGTCAGTG), where X represents dA or *N*^6^-GA-Ade (named ND30dA or *N*^6^-GA-dA30, respectively), were mixed with the 5′-[^32^P]-labeled 15-mer [d(CACTGACTGTCAAAA)] or 10-mer [d(CACTGACTGT)] primers at a molar ratio of 1.25:1 to ensure complete annealing of the labeled primer. The oligonucleotides were annealed by sequential incubations at 65 °C for 10 min, 37 °C for 10 min, and room temperature for 10 min. The 5′-[^32^P] primer-template hemiduplex (1.0 pmol) was then incubated with the catalytic fragment of human DNA polymerase ε (Enzymax; 0.2 pmol), in a solution (10 μl) containing 40 mM Tris-HCl (pH 7.5), 10 mM MgCl_2_, 100 μM each dNTPs, 10 mM dithiothreitol, and 250 μg/ml bovine serum albumin, at 37 °C for 15 min. For the time-course assay, 2.5 μl aliquots were collected from the reaction mixture at 30, 60, 120, 300, and 900 s. The reactions were terminated by adding an equal volume of 98% formamide/10 mM EDTA, followed by heat denaturation. The reaction products were separated on a 20% polyacrylamide/8 M urea gel and visualized by autoradiography, using a Typhoon FLA 9500 imager (GE Healthcare).

### Intracellular site-specific mutagenesis assay

The shuttle vector carrying a single *N*^6^-GA-dA at a specific position and the corresponding control vector were constructed as previously described (5). Briefly, the ND30dA (control) or *N*^6^-GA-dA30 oligonucleotide was annealed with the single-stranded pMTEX-GA2 vector. Complementary strand synthesis and subsequent circularization were performed using T4 DNA polymerase (TaKaRa Bio) and T4 DNA ligase (New England Biolabs). The covalently closed circular DNA was purified by cesium chloride-ethidium bromide density gradient ultracentrifugation, dialyzed against sterilized TE buffer, and concentrated using an Amicon Ultra 100 kDa filter (Merck Millipore). The shuttle vector (0.5 μg) was transfected into 2.5 × 10^5^ XP4PASV cells, which had been seeded in a 25 cm^2^ tissue culture flask 1 day before transfection. The vector was allowed to replicate in the cells for 48 h. Progeny plasmids were recovered by the method of Hirt (36) and treated with DpnI (New England Biolabs) to digest the persisting input vector. *Escherichia coli* NEB10β (*araD139* Δ(*ara-leu*) *7697 fhuA lacX74 galK* (Φ80 Δ(*lacZ*)*M15*) *mcrA galU recA1 endA1 nupG rpsL* (*Str*^*R*^) Δ [*mrr-hsdRMS-mcrBC*]; New England Biolabs) was transformed with the resulting progeny plasmids and plated on 1× YT agar plates containing 100 μg/ml carbenicillin (Sigma-Aldrich) and 50 μg/ml blasticidin S (Kaken Pharmaceutical). Carbenicillin- and blasticidin S-resistant clones were subjected to colony-direct PCR, and the PCR products were subsequently digested by FastDigest SmiI (Thermo Fisher Scientific). Replication efficiency was determined by the ratio of SmiI-resistant clones to total clones analyzed, and that of the control plasmid was set as 100%. SmiI-resistant PCR products were subjected to Sanger sequencing, performed at FASMAC, to determine mutation frequency. All experiments were performed in triplicate. The 95% exact binomial confidence interval for the mutation frequency was calculated using the Clopper-Pearson method.

### Statistical analyses

Replication efficiency and mutation frequency of *N*^6^-GA-dA were compared with the controls by a two-tailed unpaired *t* test with Welch’s correction using Prism 8 (GraphPad Software). *p* Values <0.05 were considered statistically significant. Data are presented as mean ± standard deviation.

## Data availability

All the data described in this study are contained within the article.

## Supporting information

This article contains supporting information (Figs. S1–S14).

## Conflict of interest

The authors declare that they have no conflicts of interest with the contents of this article.
