# Supplementary material for: Chemical and biological characterization of glycidamide-adducted adenine in DNA
Source: J Biol Chem. 2025 Nov 4;301(12):110898. doi: 10.1016/j.jbc.2025.110898 (PMC12769786; doi:10.1016/j.jbc.2025.110898)
Supplement: Supplementary Data [file mmc1.pdf]

Supplementary Data

Chemical and biological characterization of glycidamide-adducted adenine in DNA

Jun-ichi Akagi[1,*], Ryota Yamaguchi[2], Yumi Miyake[3,4], Masayuki Yokoi[5], Kaoru Sugasawa[5] and Shigenori Iwai[2,5,6,*]

[1] Division of Pathology, National Institute of Health Sciences, 3-25-26 Tonomachi, Kawasaki-ku, Kawasaki, Kanagawa 210-9501, Japan

[2] Division of Chemistry, Graduate School of Engineering Science, Osaka University, 1-3 Machikaneyama, Toyonaka, Osaka 560-8531, Japan

[3] Forefront Research Center, Graduate School of Science, Osaka University, 1-1 Machikaneyama, Toyonaka, Osaka 560-0043, Japan

[4] Core Facility Center, Osaka University, 1-2 Machikaneyama, Toyonaka, Osaka 560-0043, Japan

[5] Biosignal Research Center, Kobe University, 1-1 Rokkodai, Nada-ku, Kobe, Hyogo 657-8501, Japan

[6] Frontiers of Innovative Research in Science and Technology, Konan University, 7-1-20 Minatojima-Minamimachi, Chuo-ku, Kobe, Hyogo 650-0047, Japan

Table S1. DNA templates and primers used in the primer extension assay.

**Name**	**Sequence (5′→3′)**	**Notes / Modifications**
Template 30-mer (ND30dA)	5′-CTCGTCAGCATTTT**A**TTTTGACAGTCAGTG-3′	
Template 30-mer (N6-GA-dA30)	5′-CTCGTCAGCATTTT**X**TTTTGACAGTCAGTG-3′	X = N^6-GA-dA
Primer 15-mer	5′-CACTGACTGTCAAAA-3′	5′-[^{32}P]-labeled
Primer 10-mer	5′-CACTGACTGT-3′	5′-[^{32}P]-labeled

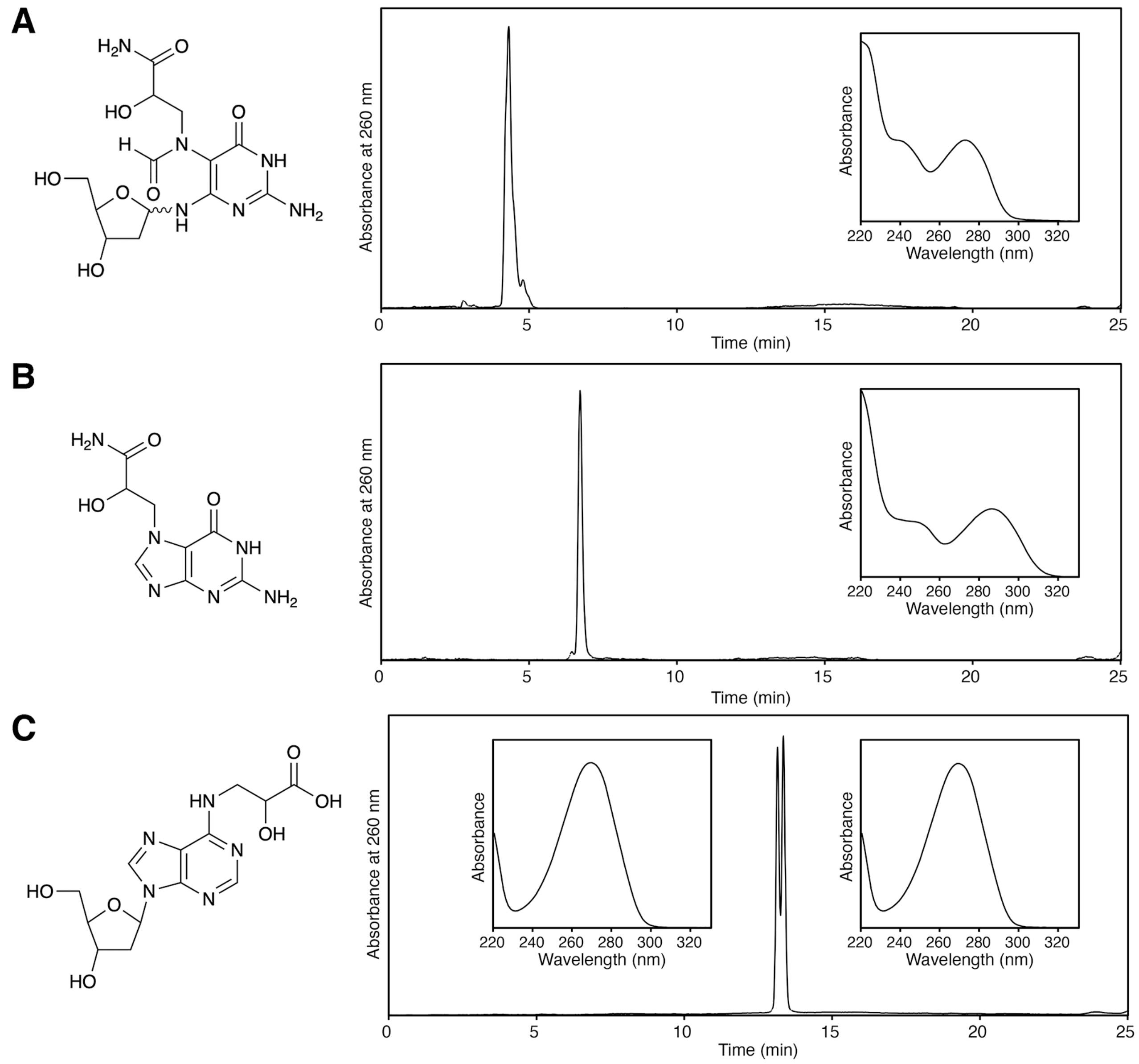

Figure S1. GA adducts prepared for the LC-MS analysis. (**A**) GA-FAPy-dG, (**B**) N7-GA-Gua, and (**C**) N^6-GA-dA. The chemical structures, HPLC chromatograms, and UV-absorption spectra of the HPLC peaks are shown. GA-FAPy-dG is shown in the form of the furanose structure, but a nucleoside bearing a FAPy derivative as a base is a mixture of the α- and β-anomers of the furanose and pyranose structures (ref. 16). The difference in the retention time of N^6-GA-dA between panel **C** and the other figures is due to the buffer, 10 mM ammonium acetate or 0.1 M TEAA, used for the eluent.

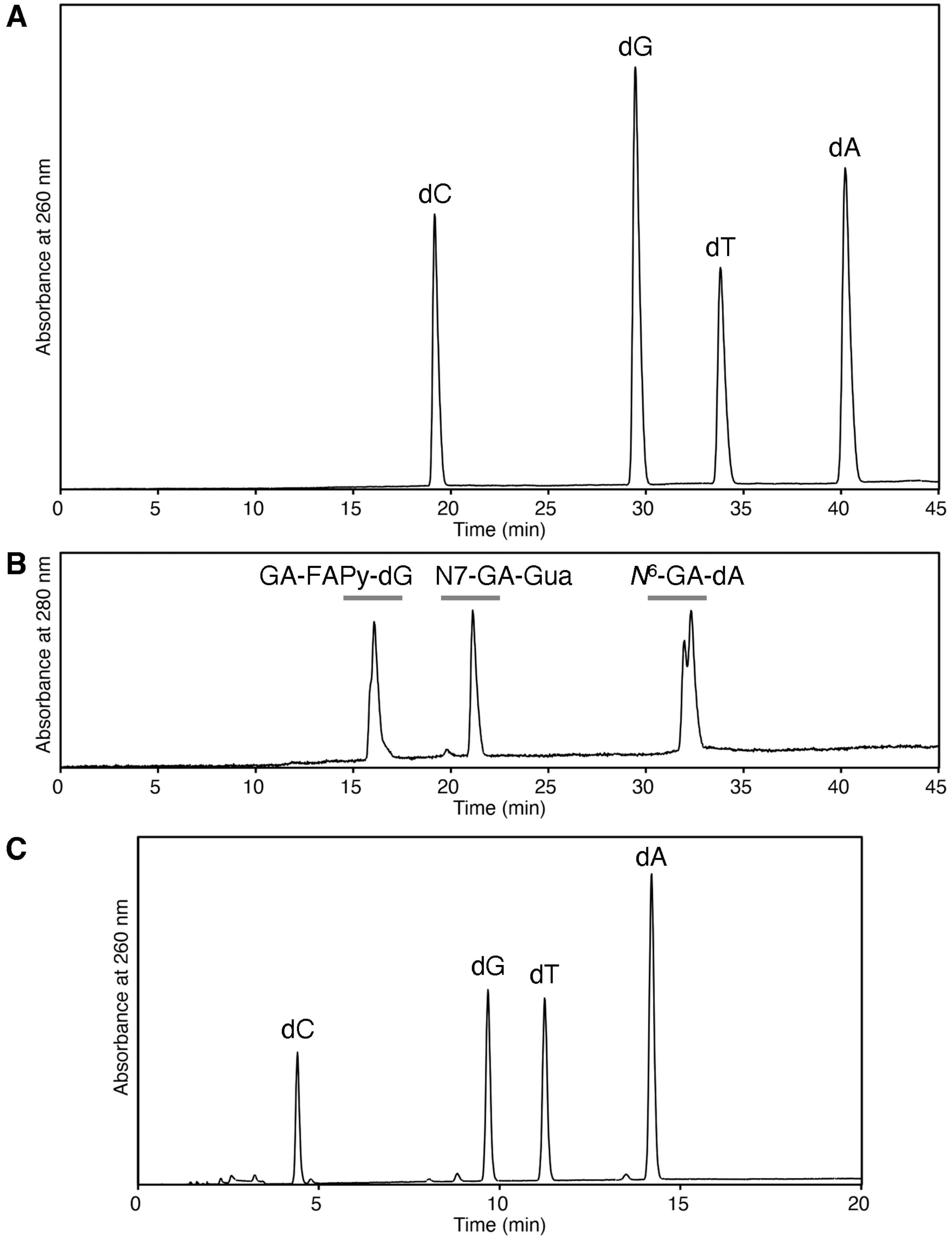

Figure S2. Sample preparation for the detection of the GA adducts in genomic DNA by LC-MS. (**A** and **B**) Preliminary experiments before the sample preparation. Canonical nucleosides (**A**) and the standard GA adducts (**B**) were analyzed on a semi-preparative column to determine their retention times. The elution conditions are the same as those used for the fractionation of the nuclease/phosphatase-treated DNA. The eluates in the ranges indicated by gray bars were collected

in the fractionation of the actual sample. (**C**) Confirmation of the complete degradation of the genomic DNA on an analytical column. Genomic DNA obtained from GA-treated XP2OSSV cells was incubated with DNase I, phosphodiesterase I, and phosphatase, and a small aliquot of the reaction mixture was injected into the HPLC column. Since the amounts of the GA adducts were too small, they could not be detected by UV absorption. Because the column and the elution conditions used in panel **C** are different from those used in panels **A** and **B**, the retention times differ.

A

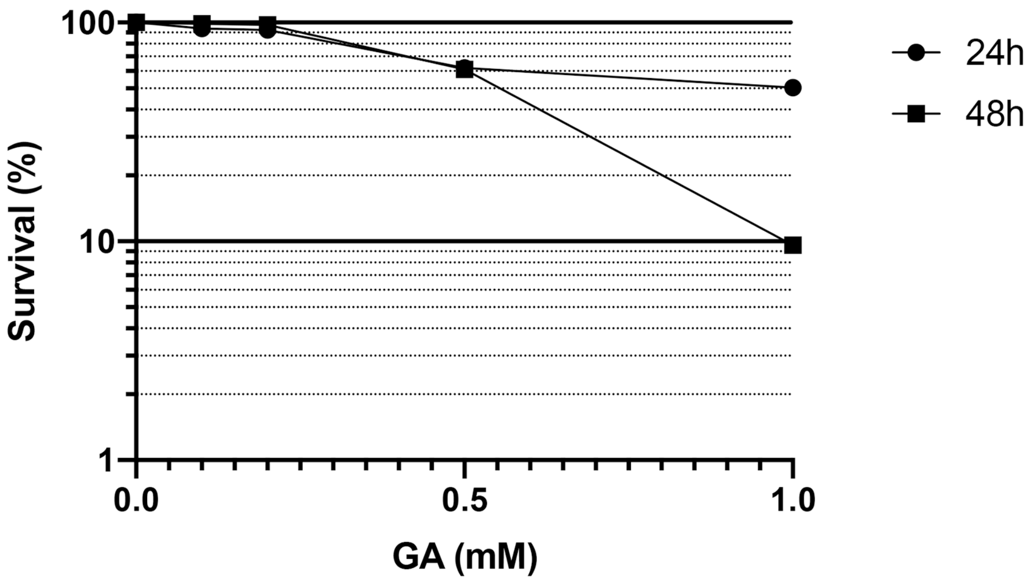

B

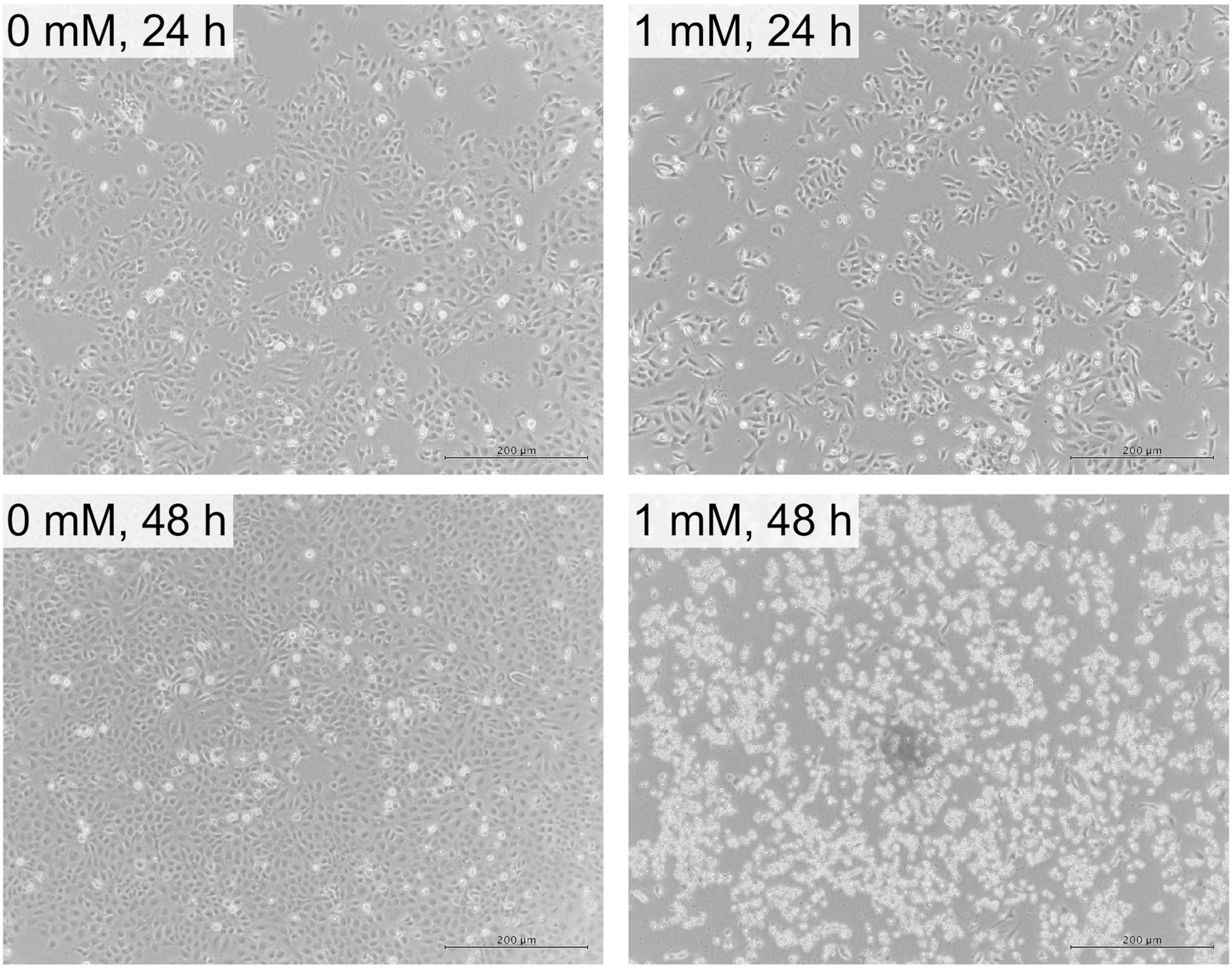

Figure S3. (**A**) Cellular survival of XP2OSSV cells following treatment with GA at the indicated concentrations and time points. (**B**) Representative phase-contrast images of GA-treated or untreated cells after 24 or 48 h.

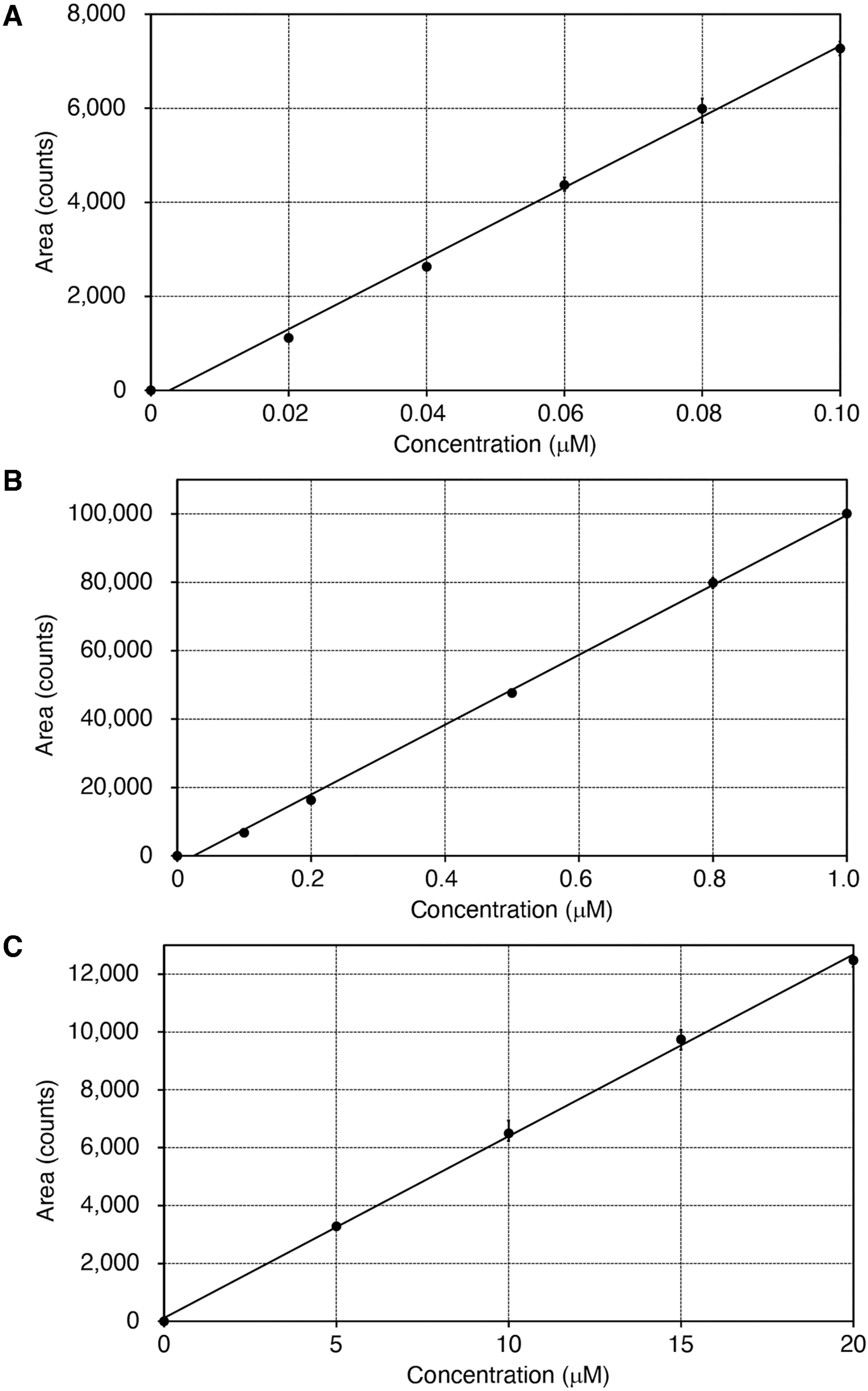

Figure S4. Calibration curves to determine the amounts of *N*[6]-GA-dA (**A**), N7-GA-Gua (**B**), and GA-FAPy-dG (**C**).

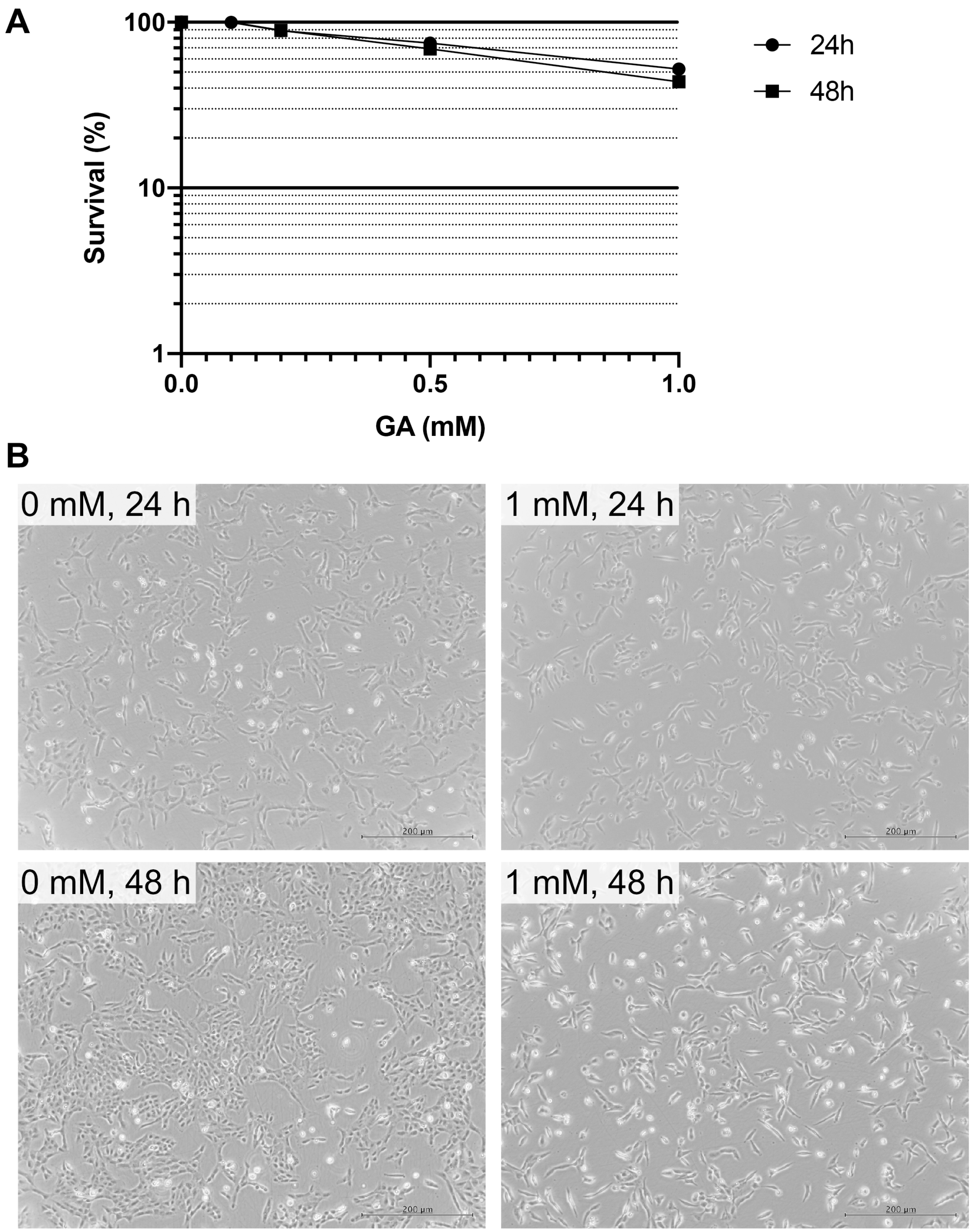

Figure S5. (**A**) Cellular survival of WI-38 VA13 cells following treatment with GA at the indicated concentrations and time points. (**B**) Representative phase-contrast images of GA-treated or untreated cells after 24 or 48 h.

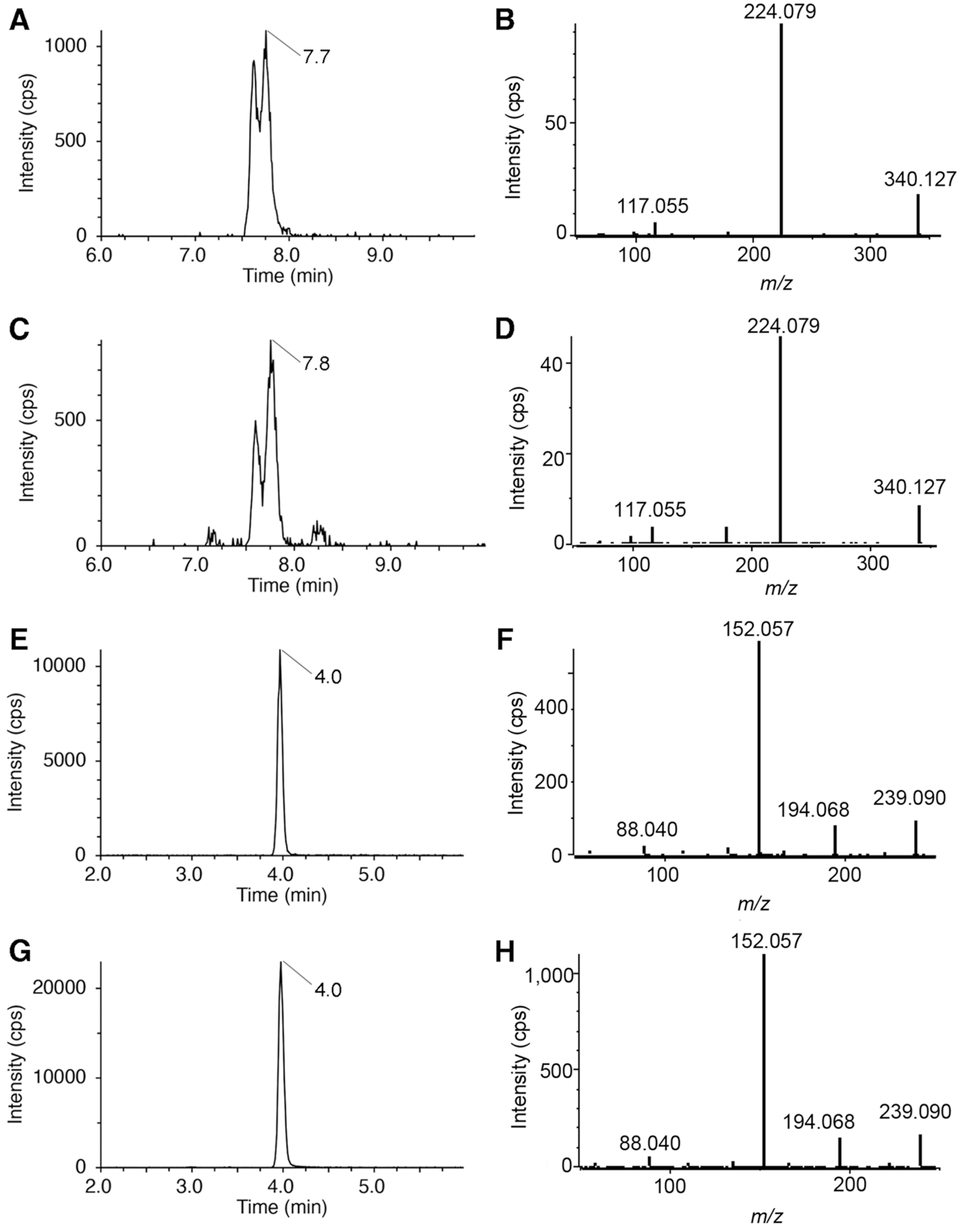

Figure S6. LC-MS analysis of the adducts formed in GA-treated NER-proficient WI-38 VA13 cells. Extracted ion chromatograms obtained from the TOF experiments (left panels) and product ion spectra acquired in the product scan experiments (right panels) are shown. (**A** and **B**) Standard N^6-GA-dA (calculated *m*/*z* 340.1252), (**C** and **D**) fraction III-2, (**E** and **F**) standard N7-GA-Gua

(calculated *m*/*z* 239.0887), (**G** and **H**) fraction II-2. The ion chromatograms were extracted at the *m*/*z* values of 340.125 (**A** and **C**) and 239.089 (**E** and **G**).

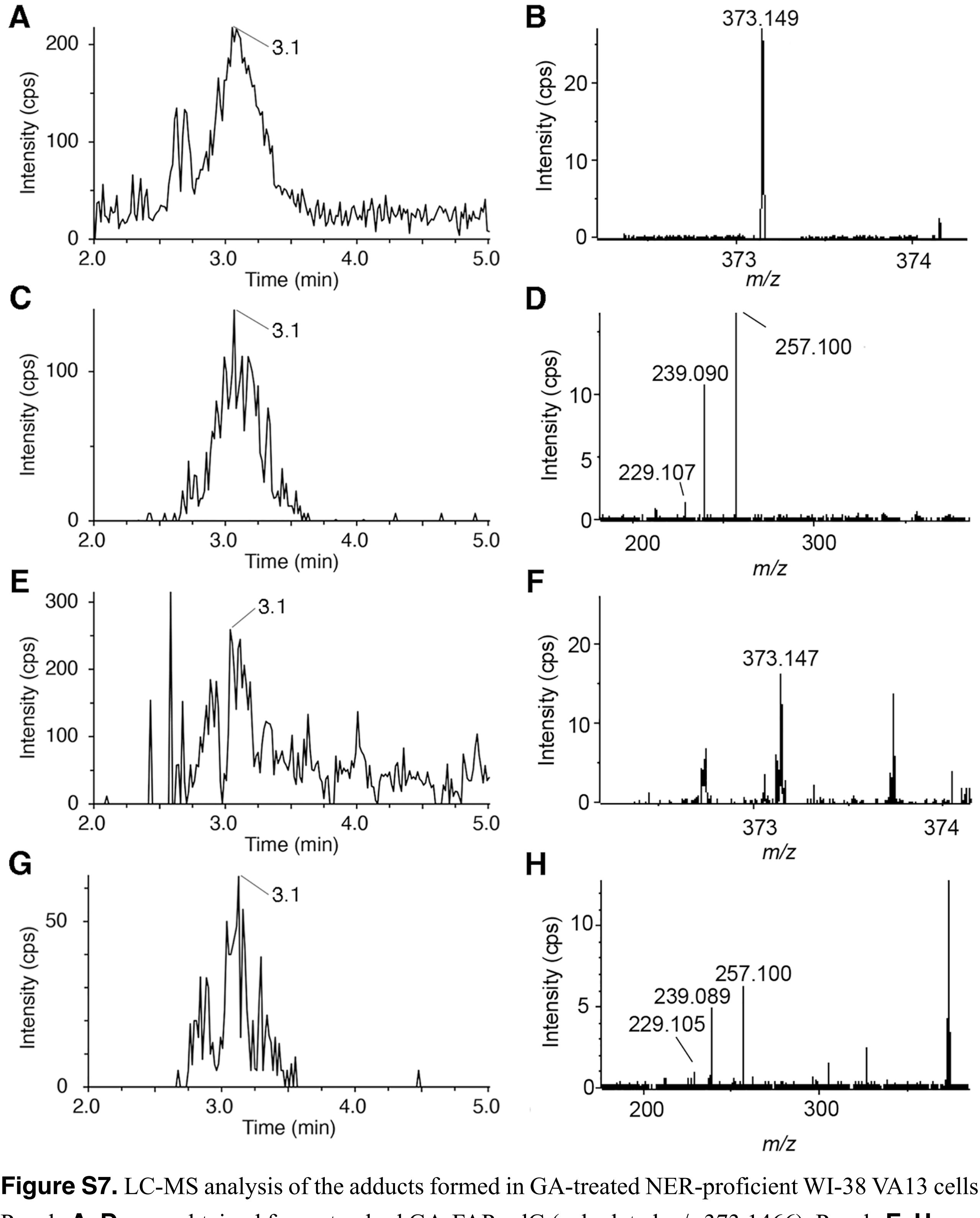

Figure S7. LC-MS analysis of the adducts formed in GA-treated NER-proficient WI-38 VA13 cells. Panels **A**–**D** were obtained from standard GA-FAPy-dG (calculated *m*/*z* 373.1466). Panels **E**–**H** were obtained from fraction I-2. (**A** and **E**) Extracted ion chromatograms (XICs) at the *m*/*z* values of 373.147 obtained from the TOF experiments, and (**C and G**) XICs at the *m*/*z* values of 257.099 obtained from the product scan experiments, are shown. Mass spectra (**B** and **F**) were obtained from the peak at 3.1 min in the XICs (**A** and **E**). Product ion spectra (**D** and **H**) were obtained from the peak at 3.1 min in the XICs (**C** and **G**).

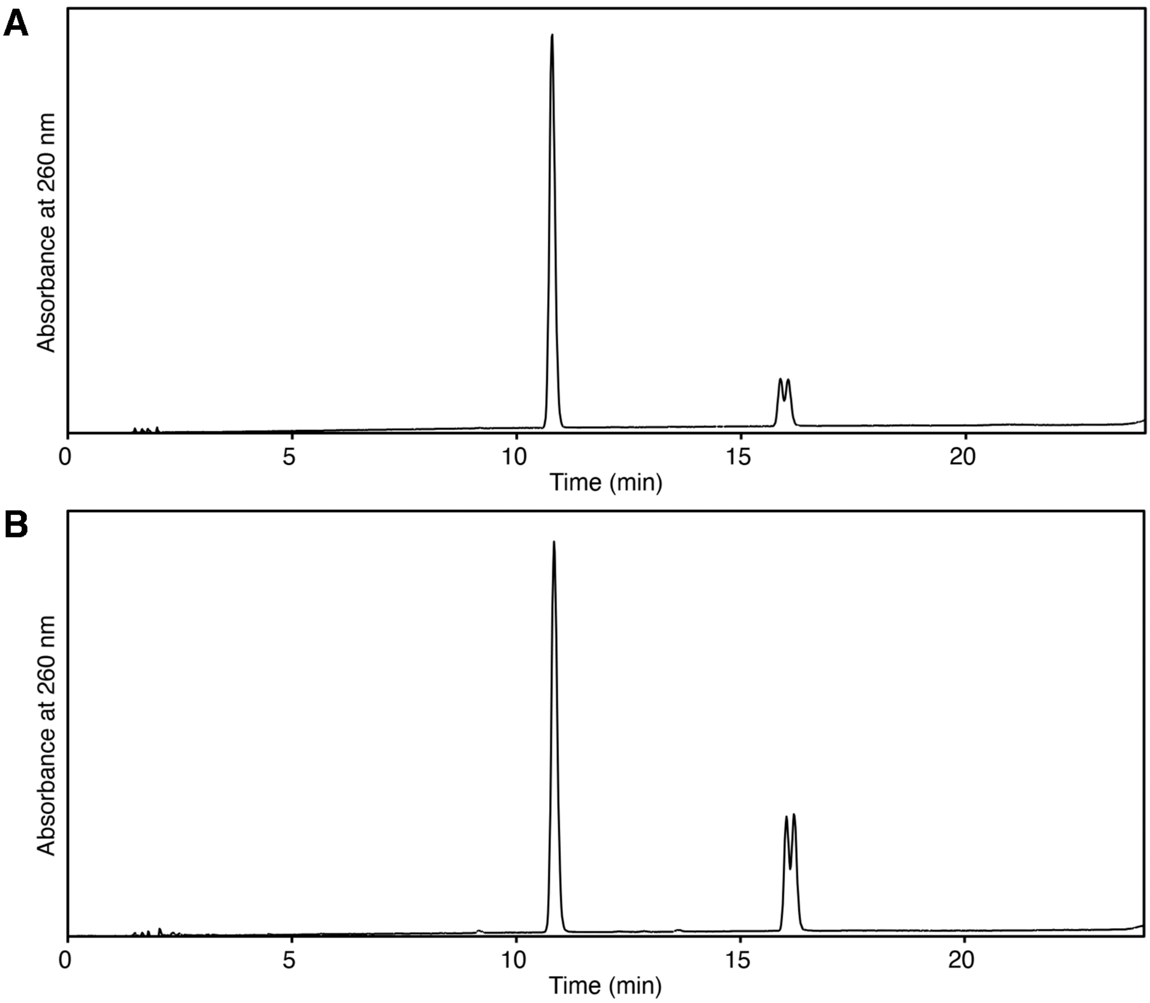

Figure S8. Analysis of the 9-mer containing N^6-GA-dA. (**A**) HPLC analysis of the nucleosides obtained by degradation of the 9-mer oligonucleotide with S1 nuclease, phosphodiesterase I, and phosphatase. The large peak with a retention time of 10.8 min is thymidine. (**B**) Co-injection of the degradation products with N^6-GA-dA.

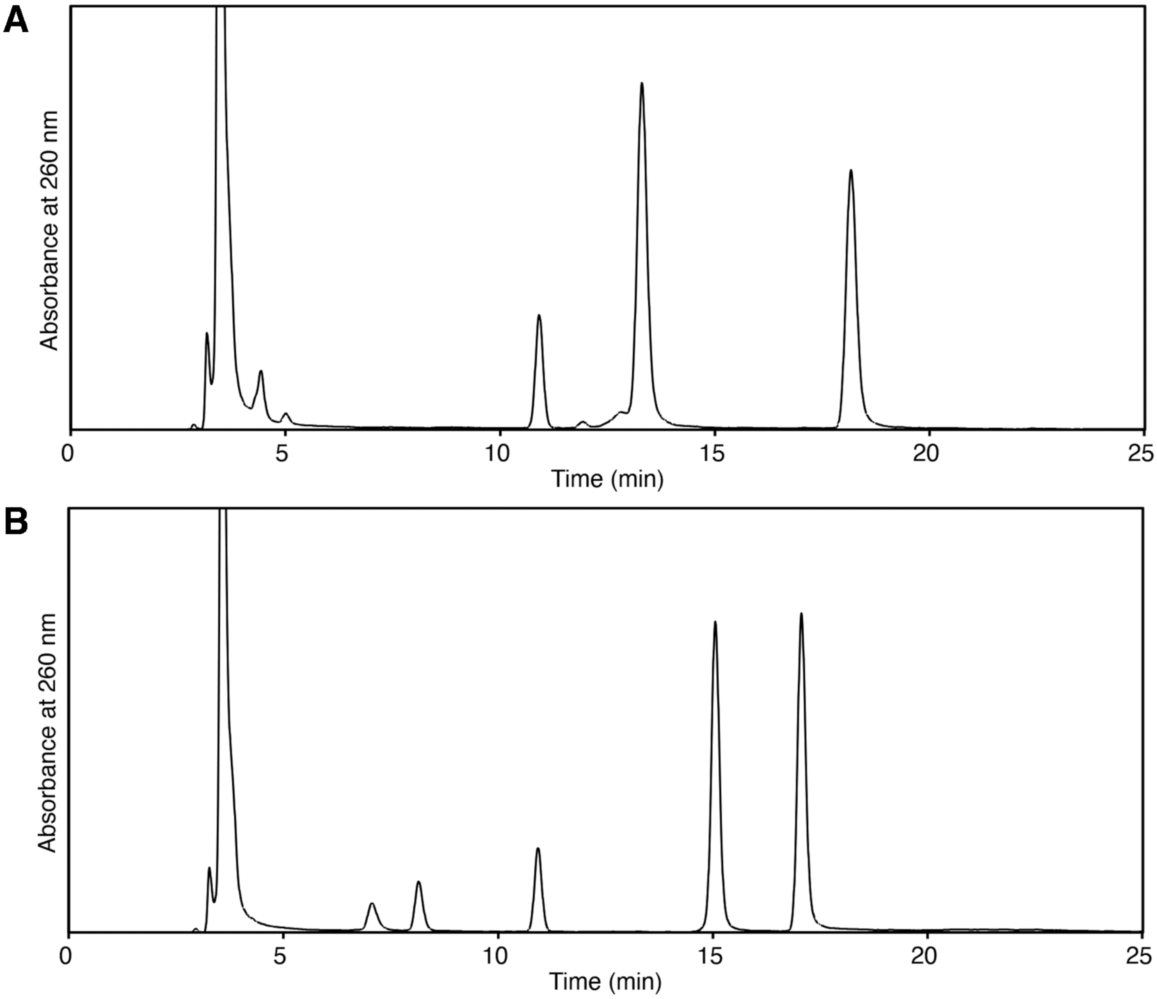

Figure S9. Preparation of the 21-mer (**A**) and the 30-mer (**B**) containing N^6-GA-dA. The ligation mixtures were analyzed by reversed-phase HPLC under heat-denaturing conditions. The peak with a retention time of 10.9 min is attributed to DNA ligase, and those at 13.3 min (**A**) and 15.1 min (**B**) are the 21-mer splints used for the ligation. The peaks with the longest retention times were collected as the desired products.

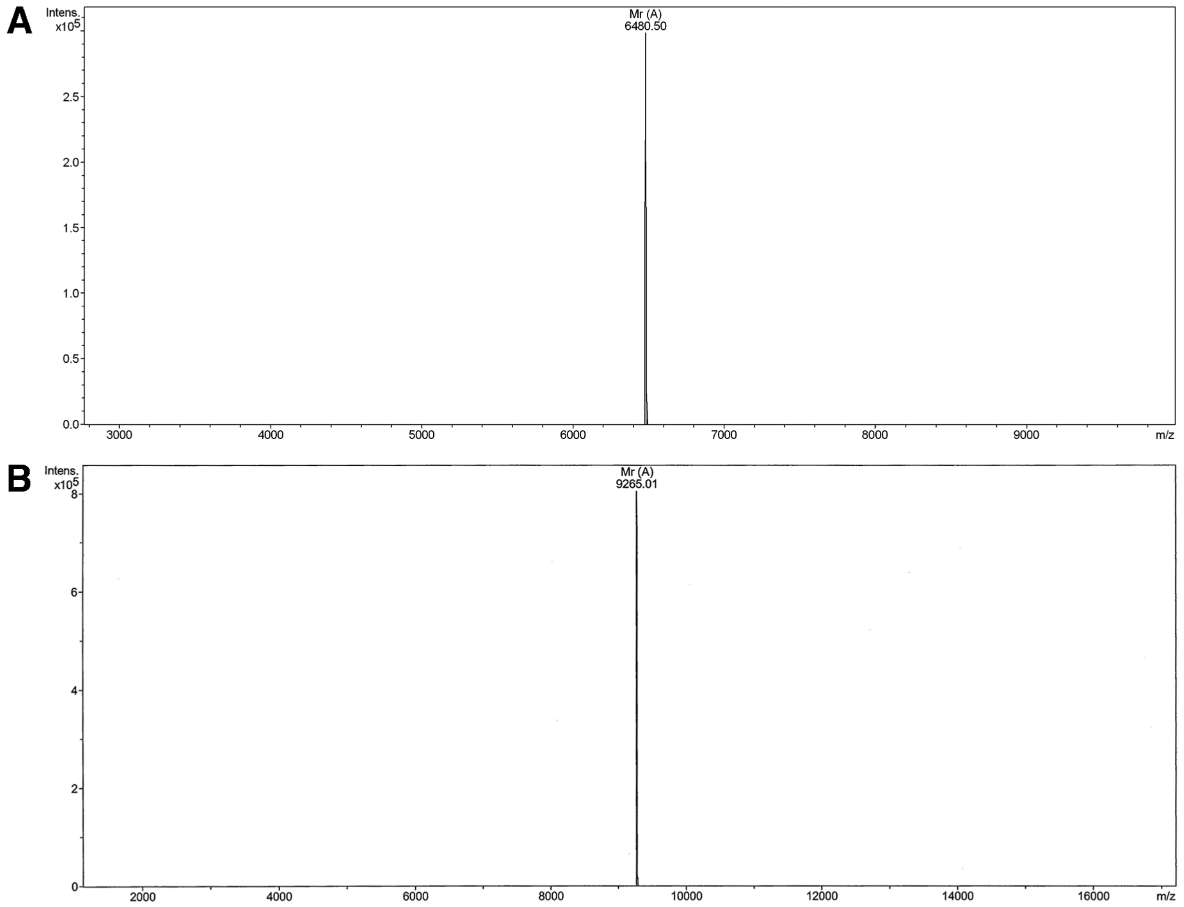

Figure S10. Analyses of the N^6-GA-dA-containing 21-mer (**A**) and 30-mer (**B**) by mass spectrometry. The calculated molecular weights are 6481.25 and 9266.05, respectively.

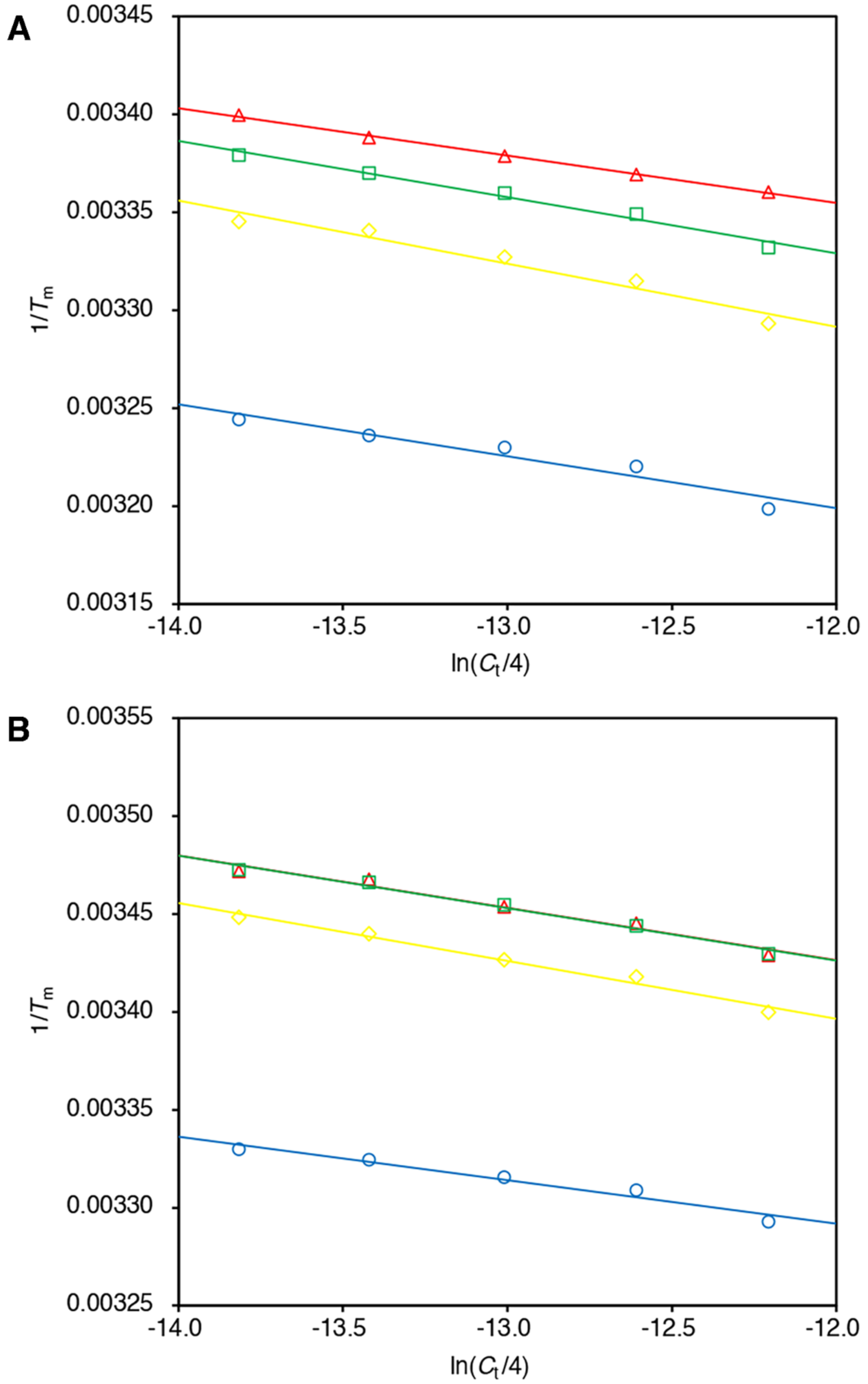

Figure S11. Plots of $1/T_m$ versus $\ln(C_t/4)$ for the duplexes, d(GCGTACTTTTXTTTTCATGCG) · d(TGAAAAYAAAAGT). (**A**) X = A. (**B**) X = N^6-GA-Ade. Y represent A (red), G (yellow), C (green), or T (blue).

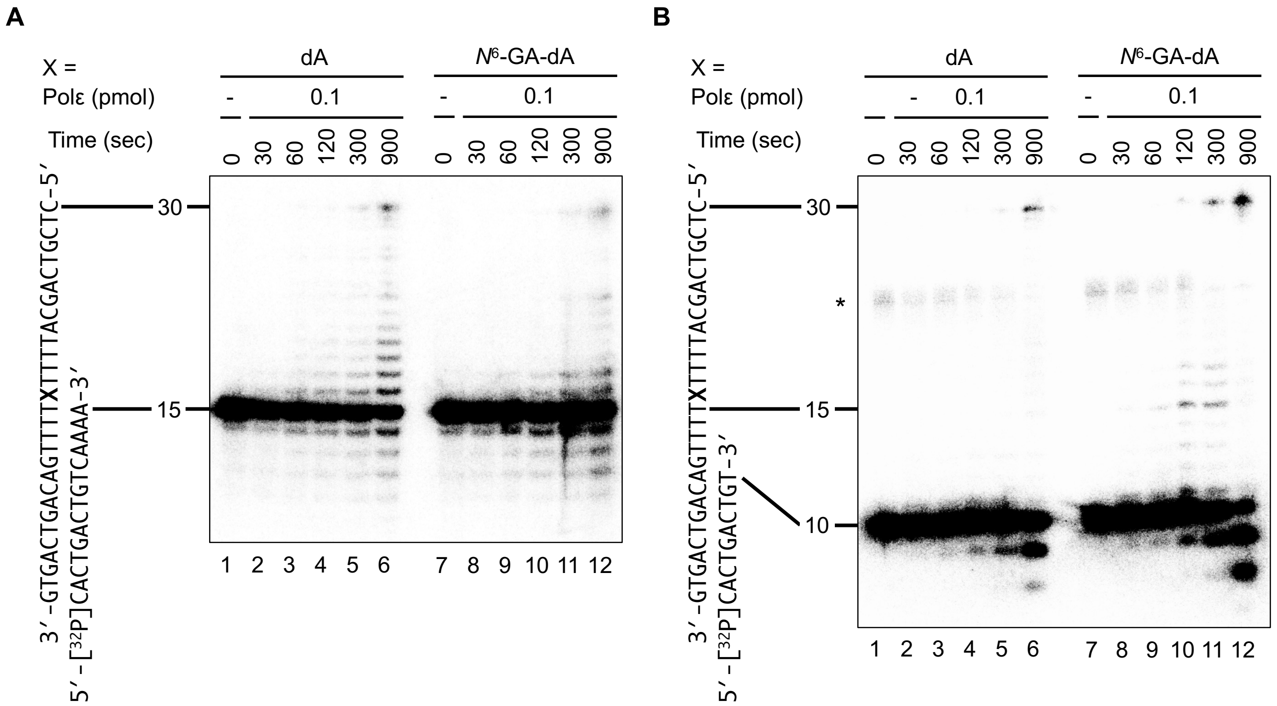

Figure S12. Time-course primer extension assays using 30-mer oligonucleotides, d(CTCGTCAGCATTTTXTTTTGACAGTCAGTG), where X represents dA or N^6-GA-dA, as templates. The [^{32}P]-labeled complementary 15-mer [d(CACTGACTGTCAAAA), **A**] and 10-mer [d(CACTGACTGT), **B**] primers were annealed to the 30-mer templates. The primer–template substrates were incubated in the presence or absence of the catalytic fragment of human Polε at 37°C for the indicated time points. The reaction mixtures were subjected to denaturing PAGE and visualized by autoradiography. *, minor contaminant contained in the 10-mer primer.

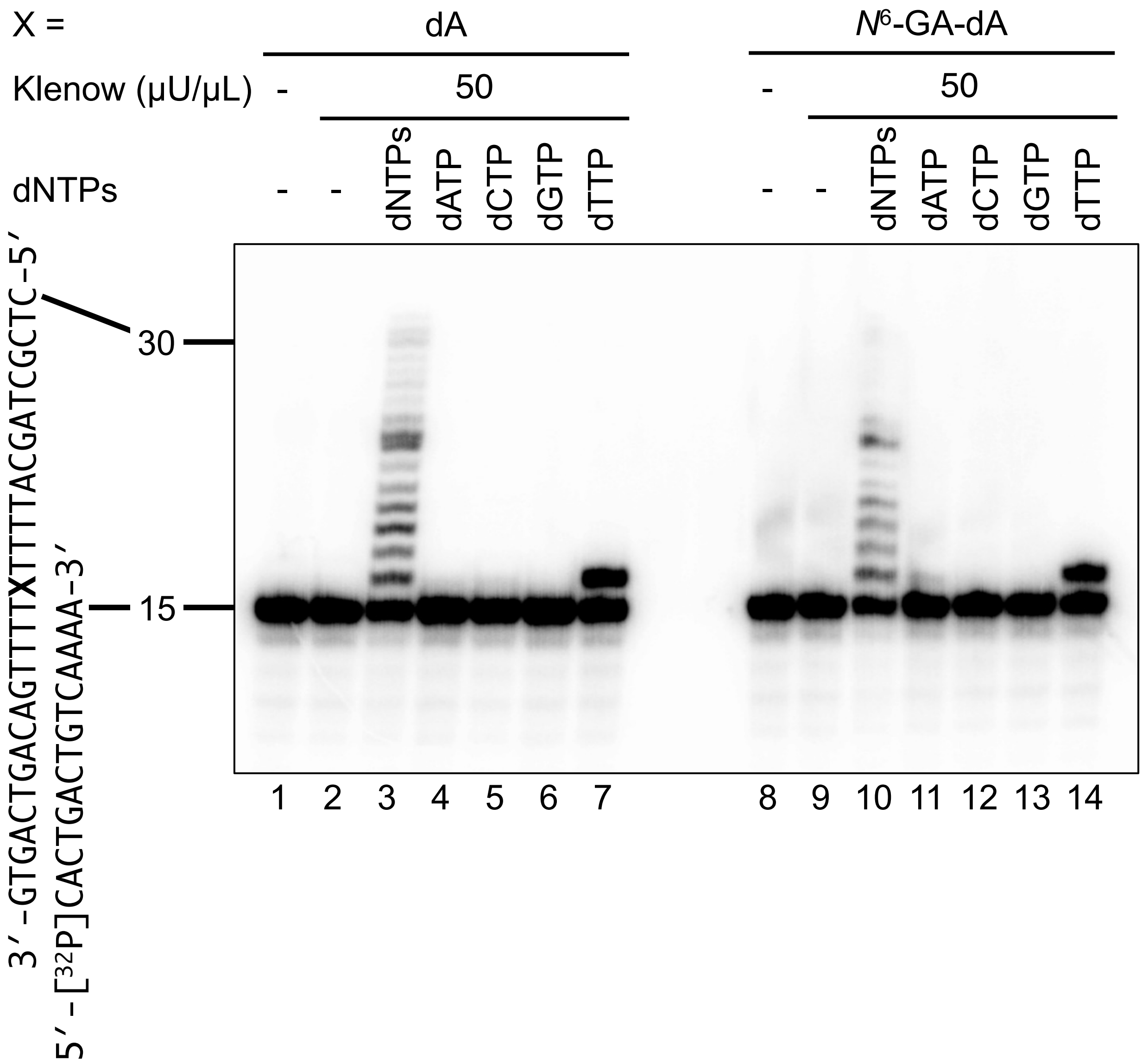

Figure S13. Single nucleotide incorporation assay using the Klenow fragment. The [^{32}P]-labeled 15-mer primer was annealed to the 30-mer templates containing dA or N^6-GA-dA at the 16th position (denoted as X). The primer–template substrates were incubated with the Klenow fragment in the presence of all four deoxynucleotide triphosphates (dNTPs) or individual dATP, dCTP, dGTP, or dTTP at 37°C for 15 min. The reaction mixtures were subjected to denaturing PAGE and visualized by autoradiography.

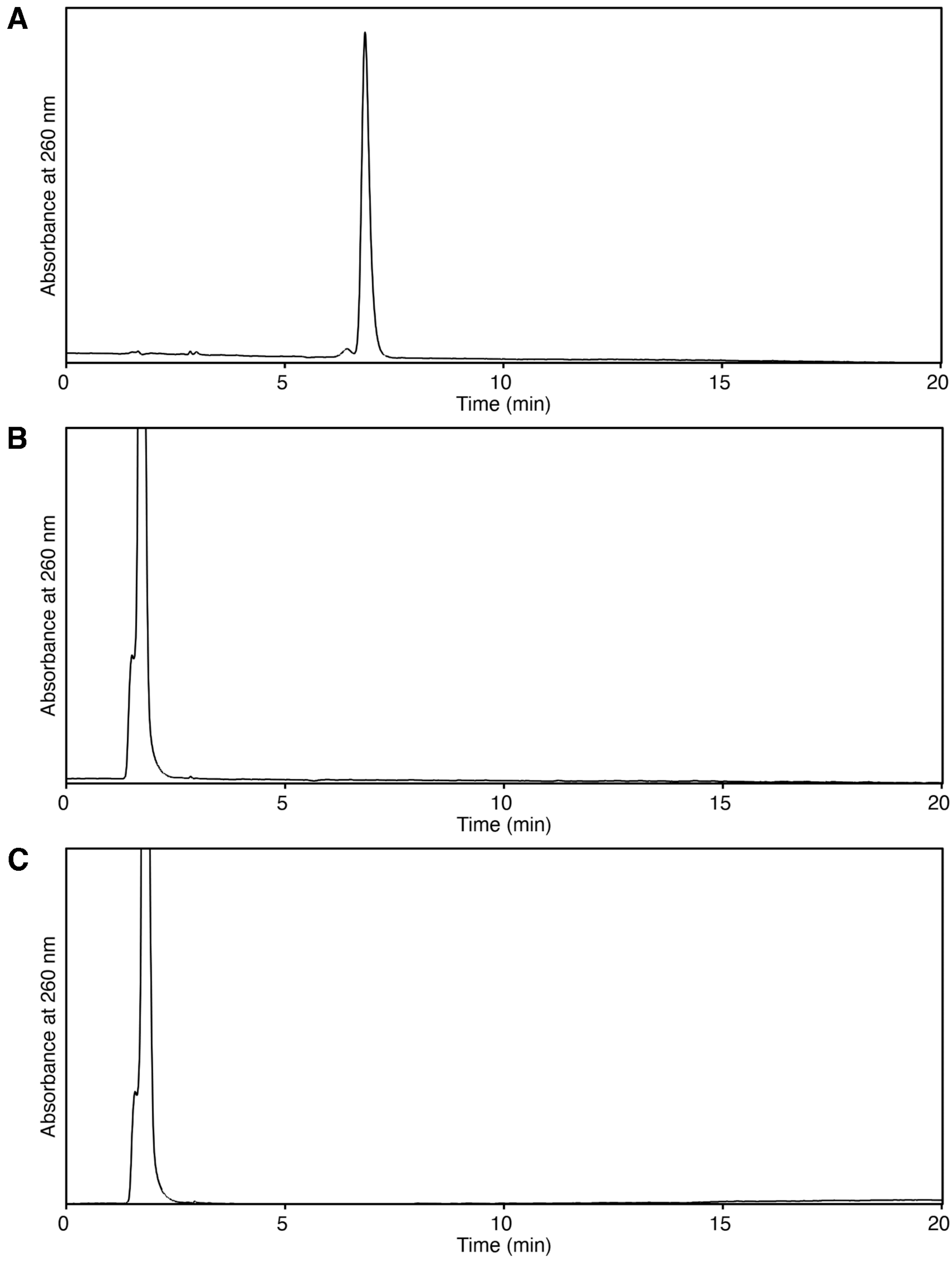

Figure S14. Loss of N7-GA-Gua during the DNA purification procedure. N7-GA-Gua (**A**) and the eluates from the DNA-purification columns of the QIAGEN DNeasy Blood & Tissue Kit, without (**B**) and with (**C**) loading of N7-GA-Gua, were analyzed under the same conditions as those used in Figure S1.
